# Effectiveness of eHealth Interventions in Alleviating Burden on Informal Caregivers of People With Dementia: Systematic Review and Meta-Analysis of Randomized Controlled Trials

**DOI:** 10.2196/78568

**Published:** 2026-06-03

**Authors:** Min Liang, Jinxia Rong, Crina Grosan, Xiyi Wang

**Affiliations:** 1 School of Nursing, Shanghai Jiao Tong University, 1 Banxia Road, Pudong New Area District, Shanghai, 201318, China, 86 19101751926; 2 School of Nursing, Fudan University, Shanghai, China; 3 Division of Digital Health and Applied Technology Assessment, Florence Nightingale Faculty of Nursing, Midwifery & Palliative Care, King’s College London, London, United Kingdom; 4 Department of Nursing, Renji Hospital, Shanghai Jiao Tong University School of Medicine, Shanghai, China

**Keywords:** caregiver burden, dementia, meta-analysis, systematic review, eHealth

## Abstract

**Background:**

Informal caregivers of people with dementia frequently experience substantial psychological burden, including elevated stress and depressive symptoms. eHealth interventions have emerged as a scalable solution to support caregivers. However, their effectiveness and the influence of intervention characteristics remain unclear.

**Objective:**

This study aimed to evaluate the effectiveness of eHealth interventions in reducing caregiver burden and depressive symptoms among informal caregivers of people with dementia, and to explore potential moderators of intervention effectiveness, including delivery modality, human support, and intervention duration.

**Methods:**

This systematic review and meta-analysis was conducted in accordance with PRISMA (Preferred Reporting Items for Systematic Reviews and Meta-Analyses) 2020 and PRISMA-S (Preferred Reporting Items for Systematic Reviews and Meta-Analyses—Search extension) guidelines. Eight electronic databases and clinical trial registries were searched from inception to March 10, 2026. Eligible studies were randomized controlled trials involving informal caregivers of people with dementia receiving interactive eHealth interventions compared with usual care or inactive controls. Study selection, data extraction, and risk-of-bias assessment were conducted independently by 2 reviewers (ML and JXR). Meta-analyses were conducted using a random-effects model with the Hartung-Knapp-Sidik-Jonkman adjustment. Heterogeneity was assessed using the *I*^2^ statistic, and 95% prediction intervals (PIs) were calculated for the primary analyses. The certainty of evidence was evaluated using GRADE (Grading of Recommendations, Assessment, Development, and Evaluation) criteria.

**Results:**

Thirty-five trials (N=3388) were included. eHealth interventions were associated with a statistically significant reduction in caregiver burden (*k*=35, standardized mean difference [SMD] −0.26, 95% CI −0.42 to −0.10; *P*=.002; τ^2^=0.16, *τ*=0.40; 95% PI −1.10 to 0.58) and a modest reduction in depressive symptoms (*k*=23, SMD −0.27, 95% CI −0.53 to −0.01; *P*=.04; τ^2^=0.31, *τ*=0.55; 95% PI −1.45 to 0.91). While average effects were statistically significant, wide PIs indicate substantial between-study heterogeneity and variability in real-world effectiveness. Subgroup analyses suggested that short-term (≤8 weeks) intervention was associated with borderline significantly stronger effects for caregiver burden, while human-supported and mobile-based interventions demonstrated larger point estimates with substantial heterogeneity. Meta-regression indicated that caregiver age was a potential moderator (*P*=.05) in univariate analysis, with decreasing effectiveness observed in older populations. Sensitivity analysis of 27 high-quality studies confirmed robust effects (SMD −0.31, *P*=.002). No significant small-study effects were detected (Egger test: *P*>.35). The overall certainty of evidence was moderate.

**Conclusions:**

eHealth interventions provide modest but variable benefits in reducing burden and depressive symptoms among informal caregivers of people with dementia. This review is timely, given the rapidly expanding digital dementia care and extends prior evidence using a more conservative Hartung-Knapp-Sidik-Jonkman framework with PIs, showing that effects vary from minimal to substantial. While average effects support effectiveness, wide PIs and moderate certainty evidence indicate that outcomes are context-dependent and influenced by heterogeneity and methodological limitations. Hybrid digital-human models should be prioritized to enhance consistency and real-world impact.

## Introduction

### Rationale

Dementia is a progressive neurodegenerative disorder and a major contributor to disability and dependency among older adults worldwide, representing a major and growing global public health priority [1]. Recent meta-analyses have demonstrated the substantial burden experienced by dementia caregivers, particularly in relation to psychological distress and care demands [2]. In parallel, global epidemiological evidence indicates that the prevalence and societal burden of dementia are rapidly increasing, placing escalating pressure on health and long-term care systems worldwide [3]. Estimates from the China Alzheimer Report 2025 indicate that the prevalence has reached 13 million, representing a substantial and rapidly escalating public health and socioeconomic challenge, particularly in aging societies where long-term care systems remain underdeveloped [4]. Beyond the clinical burden of dementia itself, the associated caregiving demands represent a critical yet often underrecognized component of the overall disease burden. Importantly, the burden associated with informal caregiving has been increasingly recognized as a parallel public health challenge, with substantial implications for both caregiver health and health system sustainability [2]. Despite growing recognition, effective and scalable solutions to support caregivers remain limited.

Most people living with dementia receive care in home and community settings. In China, cultural norms emphasizing family responsibility, combined with the high cost and limited availability of institutional care, mean that informal caregivers, typically spouses or adult children, provide the majority of long-term care [5,6]. Recent large-scale randomized controlled trials suggest that existing dementia care models may have limited impact on key caregiver outcomes such as burden, despite improvements in care satisfaction [7]. These caregivers frequently manage progressive cognitive decline, neuropsychiatric symptoms, and functional deterioration, often without formal training or adequate support [8]. Informal caregivers are often described as the “second invisible patients” of the dementia care system [9]. Caregiver burden is a multidimensional construct encompassing psychological distress, physical strain, social restriction, and financial pressure. Elevated caregiver burden is associated with adverse outcomes including depressive symptoms, reduced quality of life, and increased health care utilization. In addition to objective caregiving demands, psychosocial factors, such as coping resources and access to support services, also influence caregiver outcomes [10,11].

eHealth is defined by the World Health Organization as the use of information and communication technologies for health and health-related fields, including health care services, health education, and research [12]. Building on this broad conceptualization, eHealth interventions, including web-based programs, mobile apps, and telehealth services, offer advantages in terms of accessibility, flexibility, and scalability compared with traditional face-to-face interventions [13-15]. Recent systematic reviews suggest that such interventions can improve caregiver outcomes, although the magnitude and consistency of effects vary across intervention types and delivery formats [16]. However, despite recognition of caregiver burden, effective and scalable intervention strategies remain insufficient.

Although recent reviews have examined the effectiveness of eHealth interventions for dementia caregivers, findings remain inconsistent. Reported effects on caregiver burden range from moderate reductions to nonsignificant outcomes [16-19]. These discrepancies may reflect heterogeneity in study populations, variation in intervention components, and the inclusion of nonrandomized designs. Furthermore, many meta-analyses have relied on conventional random-effects models, which may underestimate uncertainty when the number of included studies is limited. More conservative methods, such as the Hartung-Knapp-Sidik-Jonkman (HKSJ) approach, are recommended in such contexts to reduce the risk of inflated type I errors [20,21]. In addition, the relative contribution of specific intervention characteristics, such as delivery modality, intervention duration, and the inclusion of human-supported guidance, remains unclear. Elucidating these factors is essential to optimize the design and implementation of scalable digital caregiver support programs, particularly in the context of the rapid expansion of digital health services.

To address these gaps, this study focuses exclusively on randomized controlled trials (RCTs) and applies more conservative statistical approaches, including the HKSJ method, to provide more robust and reliable estimates. Furthermore, this study systematically examines key intervention characteristics, including delivery modality, intervention duration, and guidance level, to identify features associated with improved effectiveness. Compared with previous reviews that included heterogeneous study designs or did not explore intervention-level moderators, this study aims to provide a more rigorous and mechanism-oriented synthesis. By identifying the specific components that drive effectiveness, this work offers practical guidance for the design, implementation, and scaling of eHealth interventions for dementia caregivers.

### Objectives

This review aimed to evaluate the effectiveness of eHealth interventions in reducing caregiver burden among informal caregivers of individuals with dementia. The secondary objective was to assess the effects of these interventions on caregivers’ depressive symptoms. An additional aim was to examine potential moderators of intervention effectiveness through subgroup analyses and meta-regression, including intervention delivery modality, intervention duration, and the presence of human-supported guidance. Understanding these factors may inform the development of more targeted and scalable digital interventions for dementia caregiver support.

## Methods

### Study Design and Registration

This systematic review and meta-analysis was conducted in accordance with the PRISMA (Preferred Reporting Items for Systematic Reviews and Meta-Analyses) 2020 statement [22], PRISMA-S (Preferred Reporting Items for Systematic Reviews and Meta-Analyses—Search extension) recommendations [23], and the Cochrane Handbook for Systematic Reviews of Interventions. The study protocol was prospectively registered with PROSPERO (ID: CRD42024625273).

### Information Sources

A comprehensive search was performed across 8 electronic databases: PubMed (via NCBI), Embase and Scopus (via Elsevier), Web of Science Core Collection (via Clarivate), CENTRAL (Cochrane Central Register of Controlled Trials), ProQuest Dissertations & Theses Global, and CINAHL and PsycINFO (via EBSCOhost). In addition, ClinicalTrials.gov was searched to identify ongoing or unpublished studies. Reference lists of included studies were manually screened to identify additional relevant studies. All databases were searched from inception to March 10, 2026. Reference lists of included studies and relevant systematic reviews were manually screened to identify additional eligible studies. No additional sources such as conference proceedings, organizational websites, or gray literature databases were searched, as the database strategy was considered sufficient to capture relevant peer-reviewed evidence. No contact was made with study authors, experts, or manufacturers for additional or unpublished data.

### Search Strategy

The literature search was conducted and reported in accordance with the PRISMA-S extension to ensure transparency and reproducibility [23]. The search strategy combined controlled vocabulary terms (eg, MeSH [Medical Subject Headings] and Emtree) and free-text keywords related to dementia, caregivers, and eHealth or mHealth interventions. The strategy was developed de novo by the research team and iteratively refined. The search was limited to English-language studies due to resource constraints for translation and to RCTs to ensure methodological rigor. No date restrictions were applied. No validated external search filters were used; instead, study design restrictions were implemented through a combination of indexing terms and keywords. The full search strategies for all databases are provided in Table S1 in Multimedia Appendix 1. Any deviations from PRISMA-S checklist items due to methodological constraints are explicitly reported in the manuscript.

### Eligibility Criteria

Eligibility criteria were determined according to the PICOS (Population, Intervention, Comparison, Outcomes, and Study Design) framework. (1) Population (P): studies were included if they involved informal caregivers (eg, family members, spouses, or friends) providing unpaid care for individuals with a clinical diagnosis of dementia. (2) Intervention (I): were defined as eHealth-related interventions (eg, web-based platforms, mobile apps, or telehealth programs) targeting informal caregivers of individuals with dementia. (3) Comparison (C): eligible comparators included usual care, waitlist controls, or active nondigital interventions (eg, printed educational materials). (4) Outcomes (O): the primary outcome was caregiver burden, assessed using validated standardized instruments such as the Zarit Burden Interview. To avoid selective reporting bias, all studies were included providing extractable data for burden, regardless of its status as a primary or secondary outcome in the original trial. If a study reported multiple burden scales, the Zarit Burden Interview was prioritized for pooling to enhance consistency. Secondary outcomes included depressive symptoms measured by validated scales such as the Center for Epidemiological Studies Depression Scale or equivalent, following the same inclusion as the primary outcome. (5) Study design (S): peer-reviewed RCTs published in English.

Studies were excluded if they (1) focused solely on formal or professional health care providers, (2) provided only passive web-based informational content without interactive components, or (3) were conference abstracts, editorials, or protocols without extractable outcome data.

### Study Selection and Data Extraction

All retrieved records were imported into EndNote (version 21; Clarivate Analytics) for deduplication using both automated and manual processes. Title and abstract were screened independently by 2 reviewers (ML and JXR). Screening was conducted in a 2-stage process to manage the high volume of retrieved records (n=7449). In the first stage, 2 reviewers (ML and JXR) independently performed a preliminary triage based on titles to remove clearly irrelevant records (eg, animal studies and unrelated medical conditions). To ensure a highly sensitive and overinclusive process, records were excluded at this stage only if both reviewers independently deemed them unequivocally unrelated to the research objective. In the second stage, all remaining records underwent a formal title-and-abstract screening process, in which both title and abstract information was considered, conducted independently by 2 reviewers (ML and JXR) in accordance with Cochrane recommendations [24]. This stage aimed to ensure an overinclusive and sensitive selection process. Any record considered potentially relevant by either reviewer was retained for full-text assessment. Full-text papers of potentially eligible studies were then assessed for inclusion. Discrepancies were resolved through discussion or consultation with a third reviewer (XYW).

Data extraction was conducted independently by 2 researchers (ML and JXR) using a standardized extraction form. Disagreements were resolved through discussion with a third reviewer (XYW). Extracted data included (1) study characteristics (author, year, and country), (2) participant demographics (age, gender, and relationship to care recipient), (3) intervention characteristics (modality, duration, guidance, and theoretical framework), and (4) outcome data (sample size, means, and SDs). For studies with multiple intervention arms, the control group was proportionally divided to prevent double-counting.

### Study Risk-of-Bias Assessment

The risk of bias for each included RCT was assessed using the Cochrane Risk of Bias 2 (RoB 2) tool. Five domains were evaluated: bias arising from the randomization process, deviations from intended interventions, missing outcome data, measurement of the outcome, and selection of the reported result. Two reviewers (ML and JXR) independently assigned a rating of “Low risk,” “Some concerns,” or “High risk” to each domain. Two reviewers (ML and JXR) conducted assessments independently, with disagreements resolved through consensus.

### Statistical Analysis and Meta-Analysis

#### Effect Size Calculation

Meta-analyses were conducted using a random-effects framework with inverse-variance weighting. Effect sizes were expressed as Hedges *g* (bias-corrected standardized mean difference [SMD]) to account for potential bias in small sample sizes and to enable pooling across studies that used different assessment scales for caregiver outcomes. All analyses were performed using R (meta package).

#### Heterogeneity and Random-Effects Model

Pooled estimates were calculated using the HKSJ method, which produces more conservative confidence intervals and reduces the risk of inflated type I error when the number of included studies is small [20]. The Hartung-Knapp adjustment was applied and explicitly labeled in the forest plots. The Sidik-Jonkman method was used to estimate between-study variance (τ²). Statistical heterogeneity was assessed using the *I^2^* statistic and the Cochran Q test. Furthermore, 95% prediction intervals (PIs) were calculated for the primary meta-analysis to reflect the expected range of true effects in future settings. PIs were not calculated for subgroup analyses due to methodological limitations [25].

#### Subgroup and Meta-Regression Analyses

Prespecified subgroup analyses were conducted to explore sources of heterogeneity, focusing on caregiver dominant type, intervention modality (Web-based, App/Instant Messaging (IM) (Mobile), or Video/Tele (Videoconferencing/[VC]), interaction type (Asynchronous, Synchronous), intervention guidance category (Self-guided vs Human-supported), comparator type, outcome hierarchy, monitoring, and intervention duration. Meta-regression was performed to examine the influence of continuous moderators, including mean age, proportion of female participants, and study attrition rate.

#### Small-Study Effects and Sensitivity Analysis

Potential small-study effects were evaluated using a contour-enhanced funnel plot and Egger linear regression test [26]. Sensitivity analyses were conducted by sequentially excluding studies assessed as having a high risk of bias to evaluate the robustness of the pooled estimates.

#### Certainty of Evidence Assessment

The overall certainty of evidence for each outcome was evaluated using the GRADE (Grading of Recommendations, Assessment, Development, and Evaluation) approach, considering risk of bias, inconsistency, indirectness, imprecision, and publication bias. The certainty of evidence was categorized as high, moderate, low, or very low.

#### Synthesis Methods and Visualization

Study characteristics were summarized in tabular form (Table 1). Forest plots were generated to present individual study effect sizes alongside pooled estimates, including SMDs, 95% CIs, and 95% PIs.

**Table 1.** Characteristics of included randomized controlled trials evaluating eHealth interventions for informal caregivers of people with dementia (abridged edition; *k*=35).

Study	Sample size (I/C)	Delivery/Duration	Intervention component	Outcome
Beauchamp et al [27], 2005 (United States)	150/149	Web/Asynch^a^/4w	Self-Guided: Multicomponent (Education + CBT^b^ skills)	Strain^c^ (CSI^d^) Dep^e^ (CES-D^f^)
Gitlin et al [28], 2010 (United States)	117/122	VC^g^/Synch/^h^24w	Human-Supported: Caregiver Training (ACT^i^ model)	Burden (ZBI^j^) Dep (CES-D)
Kwok et al [29], 2013 (China)	18/20	Mobile/Synch/12w	Self-Guided: Psychoeducation	Burden (ZBI)
Torkamani et al [30], 2014 (United Kingdom, Spain, and Greece)	27/30	Web/Asynch/24w	Self-Guided: Monitoring & Alerts	Burden (ZBI)
Kales et al [31], 2018 (United States)	26/30	Mobile/Asynch/4w	Human-Supported: Symptom Management (Tool-based)	Dep (CES-D)^c^; Burden (ZBI)
Meichsner et al [32], 2019 (Germany)	15/15	Web/Asynch/8w	Human-Supported: Online CBT Modules	Burden (VAS^k^)^c^; Dep (CES-D)^c^
Metcalfe et al [33], 2019 (United Kingdom, France, and Germany)	29/29	Web/Asynch/6w	Self-Guided: RHAPSODY Program	Burden (BSFC-10)^l^ Dep (PSS-10^m^)
Williams et al [34], 2019 (United States)	43/41	VC/Hybrid/12w	Human-Supported: Video-based Feedback	Burden (ZBI)^c^; Dep (CES-D)^c^
James et al [35], 2021 (United States)	5/5	Mobile/Asynch/2w	Self-Guided: Mind–Body + HRV^n^ Monitoring	Burden (ZBI)
Baruah et al [36], 2021 (India)	29/26	Web/Asynch/12w	Self-Guided: iSupport (Multi-module)	Burden (ZBI)^c^; Dep (CES-D)^c^
Fossey et al [37], 2021 (United Kingdom)	53/101/54	Web/Asynch/26w	Human-Supported: CBT-based Program	Dep (HADS^o^); Burden (RSS^p^)
Teles et al [38], 2022 (Portugal)	11/20	Web/Asynch/12w	Self-Guided: Online Training (iSupport)	Burden (ZBI)^c^; Dep (CES-D)
Hepburn et al [39], 2022 (United States)	96/165	VC/Synch/7w	Human-Supported: Psychoeducation + Coaching	Burden (ZBI)^c^; Dep (HADS-D)
Bodenstein [40], 2022 (Canada)	16/14	VC/Synch/8w	Human-Supported: Mindful Yoga	Burden (ZBI) Dep (PHQ-9^q^)
Han et al [41], 2023 (United States)	9/10	VC/Synch/8w	Human-Supported: ACT + Behavioral Activation	Burden (ZBI) Dep (DASS-21^r^)
Rhodus et al [42], 2023 (United States)	9/9/7	VC/Synch/6w	Human-Supported: Sensory-based Intervention	Burden (ZBI)
Rodriguez et al [43], 2023 (United States)	23/22	Mobile/Asynch/24w	Human-Supported: Digital Coaching + Monitoring	Burden (NPI-Q^s^)
Hu et al [44], 2024 (China)	30/30	Mobile/Synch/8w	Human-Supported: MBSR^t^	Burden (ZBI)^c^; Dep (DASS-21)
Salehinejad et al [45], 2024 (Iran)	24/23	Web/Asynch/8w	Self-Guided: Education + Behavioral Management	Burden (ZBI)
Xie et al [46], 2024 (China)	33/33	Web/Asynch/24w	Self-Guided: Caregiver Training (Nurse-led)	Burden (ZBI)
Jain [47], 2024 (India)	8/8	VC/Synch/3w	Human-Supported: CBT+ Mindfulness	Burden (ZBI)^c^
Song et al [48], 2024 (United States)	13/14	VC/Synch/5w	Human-Supported: CBT-I + Behavioral Activation	Burden (ZBI)
Yuan et al [49], 2025 (China)	51/55	Mobile/Synch/20w	Human-Supported: iSupport + Follow-up	Burden (ZBI)
Nguyen pilot et al [50], 2025 (Vietnam)	27/30	Mobile/Asynch/7w	Human-Supported: Psychoeducation + Discussion	Burden (ZBI); Dep (DASS-21)
Nguyen et al [51], 2025 (Vietnam)	80/81	Mobile/Asynch/7w	Human-Supported: Video Psychoeducation + Messaging	Dep (DASS-21)^c^; Burden (ZBI)
Han et al [52], 2025 (United States)	16/17	VC/Synch/10w	Self-Guided: ACT-based	Dep (PHQ-9)^c^; Burden (ZBI)
Nichols et al [53], 2025 (United States)	56/54	Mobile/Synch/12w	Human-Supported: Problem-Solving + Coping	Burden (ZBI)^c^; Dep (PHQ-9)
Stevens et al [54], 2025 (United States)	79/88	Web/Asynch/24w	Human-Supported: Psychoeducation + Skills + Network	Burden (ZBI)^c^; Dep (CES-D)
Pfaff et al [55], 2025 (Germany)	83/87	VC/Hybrid/24w	Human-Supported: Needs Assessment + Care Planning	Burden (ZBI)
Windle et al [56], 2025 (United Kingdom)	175/177	Web/Asynch/24w	Self-Guided: iSupport (5 modules)	Burden (ZBI)^c^; Dep (CES-D)^c^
Sun et al [57], 2026 (China)	98/103	Mobile/Asynch/12w	Self-Guided: Personalized Care + Consultation	Burden (ZBI)^c^; Dep (SDS^u^)
Yuan et al [58], 2026 (Singapore)	27/26	Mobile/Asynch/4w	Self-Guided: Knowledge Base + Peer Support	Dep (CES-D)^c^; Burden (ZBI)
Brijnath et al [59], 2026 (Australia)	47/46	Web/Asynch/12w	Self-Guided: WHO^v^ iSupport Lite (Multimedia)	Burden (ZBI)^c^; Dep (CES-D)
Cheng and Ng [60] 2026 (43 countries)	132/142	Web/Synch/12w	Self-Guided: Multi-app + AI^w^ Support	Dep (PHQ-9)^c^; Burden (ZBI)
Durepos et al [61], 2026 (Canada)	14/15	VC/Synch/16‐24w	Human-Supported: ACT	Burden (ZBI); Dep (DASS-21)

a Asynch: asynchronous.

b CBT: cognitive behavioral therapy.

c Primary outcome as defined in the original study.

d CSI: caregiver Strain Index.

e Dep: depression.

f CES-D: Center for Epidemiologic Studies Depression Scale.

g VC: videoconferencing.

h Synch: synchronous.

i ACT: advancing caregiver training.

j ZBI: Zarit Burden Interview.

k VAS: Visual Analogue Scale.

l BSFC-10: Burden Scale for Family Caregivers.

m PSS-10: Perceived Stress Scale.

n HRV: heart rate variability.

o HADS: Hospital Anxiety and Depression Scale.

p RSS: Relative Stress Scale.

q PHQ-9: Patient Health Questionnaire-9.

r DASS-21: Depression, Anxiety and Stress Scale.

s NPI-Q: Neuropsychiatric Inventory Questionnaire (Caregiver Distress subscale).

t MBSR: mindfulness-based stress reduction.

u SDS: Zung Self-Rating Depression Scale.

v WHO: World Health Organization.

w AI: artificial intelligence.

## Result

### Study Selection and Flow Diagram

**Figure 1.**
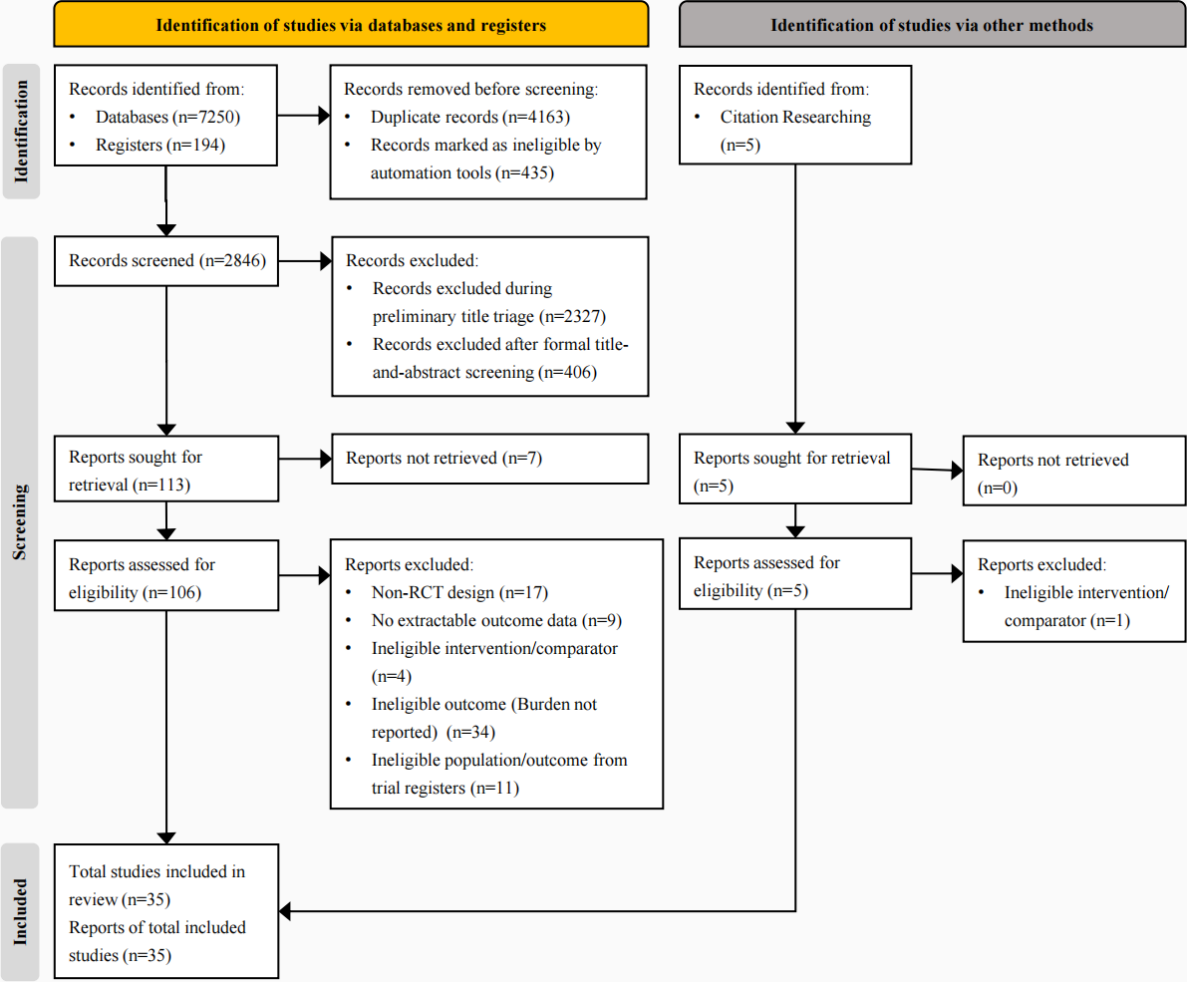
PRISMA (Preferred Reporting Items for Systematic Reviews and Meta-Analyses) 2020 flow diagram illustrating the systematic selection process of RCTs evaluating eHealth interventions for informal caregivers of people with dementia across 8 electronic databases. RCT: randomized controlled trial.

The systematic search across 8 electronic databases and registers identified 7444 records. After removing duplicates and records marked as ineligible by automation tools, 2846 records remained for screening. Following title and abstract screening, 113 reports were sought for retrieval, of which 7 could not be retrieved. A total of 106 reports from databases and registers were assessed for eligibility. In addition, 5 reports were identified through citation searching and assessed separately for eligibility. After excluding 75 reports from database and register searches and 1 report identified through citation searching, 35 RCTs were included in the review. The study selection process is detailed in the PRISMA 2020 flow diagram (Figure 1).

### Study Characteristics

The 35 included RCTs, published between 2005 and 2026, were conducted across North America, Asia, Europe, and Oceania [27-61] (Table 1; details in Table S2 in Multimedia Appendix 1 [27-61]). The trials collectively enrolled 3388 informal caregivers (intervention: n=1700; control: n=1688). Caregivers were predominantly female (mean proportion 73.06%, SD 14.73%) and were generally middle-aged to older adults, with reported mean or median ages ranging from approximately 30 to 73 years. Most participants were spouses or adult children of individuals with dementia. Interventions varied in format and duration. Early studies primarily used web-based platforms, whereas more recent trials increasingly adopted mobile or hybrid digital delivery models. Intervention duration ranged from 2 to 26 weeks. Approximately 85.71% (30/35) of interventions were explicitly grounded in established theoretical frameworks, such as the Stress and Coping Model or the World Health Organization’s iSupport program. Detailed study characteristics are presented in Table 1 and Table S2 in Multimedia Appendix 1.

### Risk-of-Bias Assessment

**Figure 2.**
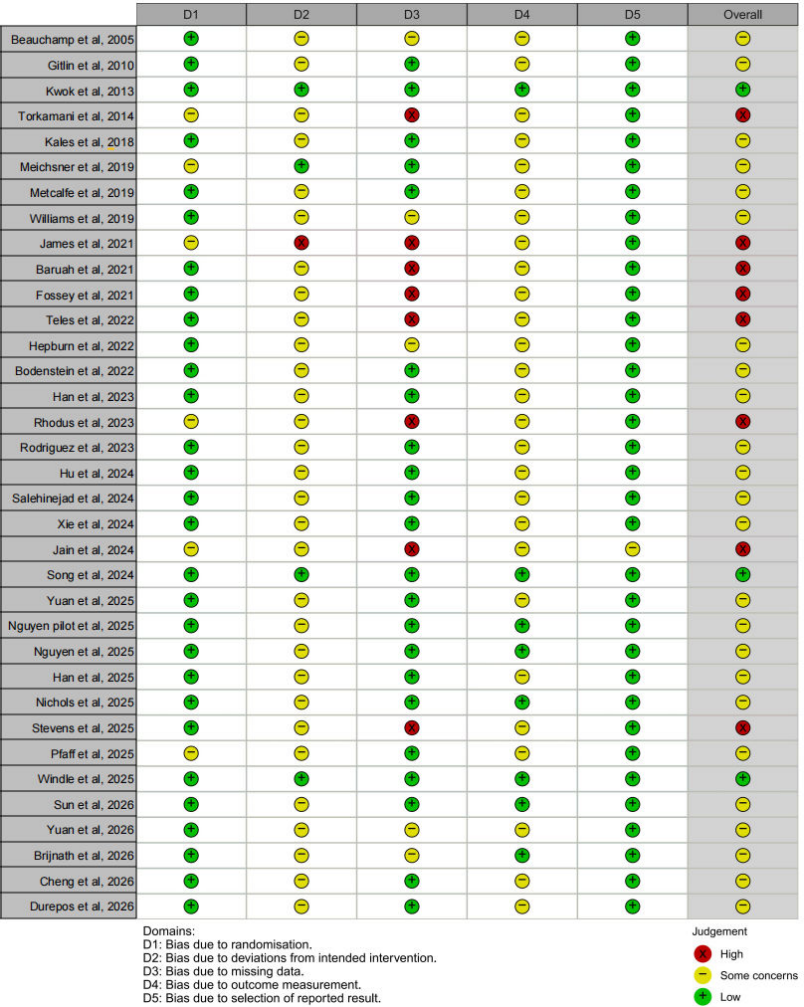
Risk-of-bias assessment for 35 randomized controlled trials using the RoB 2 tool, evaluating internal validity across 5 domains for eHealth interventions targeting dementia caregiver burden and depression [27-61].

Risk of bias was assessed using the RoB 2 tool (Figure 2 [27-61]). Three (8.6%) studies were rated as low risk, 24 (68.6%) studies had some concerns, and 8 (22.9%) studies were judged to have high risk of bias. The most common sources of bias were related to deviations from intended interventions and outcome measurement. These issues primarily reflected the inherent difficulty of blinding participants in behavioral and digital interventions and the reliance on self-reported outcome measures. Most studies showed low risk of bias for randomization and selective outcome reporting.

### Synthesis of Primary Outcome: Caregiver Burden

#### Overall Effect and PI

**Figure 3.**
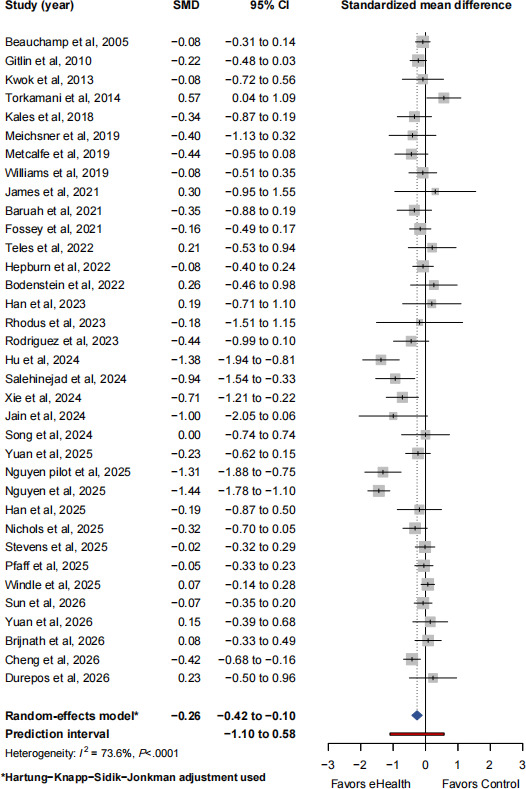
Forest plot of the effect of eHealth interventions on caregiver burden in informal caregivers of people with dementia (*k*=35), using the Hartung-Knapp-Sidik-Jonkman random-effects model. Red lines represent the 95% prediction intervals [27-61], SMD: standardized mean difference.

Thirty-five studies involving 3388 participants (intervention: n=1700; control: n=1688) were included in the meta-analysis. Using a random-effects model with the HKSJ adjustment, eHealth interventions were associated with a statistically significant reduction in caregiver burden compared with control conditions (SMD −0.26, 95% CI −0.42 to −0.10; *t*_34_=−3.32; *P*=.002; τ^2^=0.16, *τ*=0.40; 95% PI −1.10 to 0.58; Figure 3 [27-61]). Substantial heterogeneity was observed across studies (*I*² 73.6%, 95% CI 63.2%-81.0%; *Q*=128.61; *df*=34; *P*<.001). The 95% PI indicates considerable variability in the potential effects of eHealth interventions across different contexts and implementations.

#### Subgroup Analyses for Caregiver Burden

Prespecified subgroup analyses were conducted to explore the potential sources of heterogeneity (Table 2 and Figure S1 in Multimedia Appendix 1 [27-61]).

**Table 2.** Subgroup analyses of eHealth interventions on caregiver burden (*k*=35)^a^.

Variables and levels	*K*	SMD^b^	95% CI	*I* ^2^	*P*_within_
Caregiver dominant type					
Adult-child-dominant	15	−0.284	−0.583 to 0.015	84.20%	.06
Spousal-dominant	10	−0.183	−0.295 to −0.072	0.00%	*.005*
Mixed	10	−0.305	−0.720 to 0.110	74.00%	.13
Modality					
Web-based	13	−0.183	−0.410 to 0.043	62.60%	.10
Video/Tele (VC)	11	−0.086	−0.240 to 0.069	0.00%	*.24*
App/IM (Mobile)	11	−0.505	−0.914 to −0.097	85.20%	*.02*
Interaction					
Asynchronous	19	−0.292	−0.544 to −0.040	82.20%	*.03*
Synchronous	16	−0.224	−0.429 to −0.018	45.50%	*.04*
Guidance category					
Self-guided	15	−0.143	-0.348 to 0.062	57.00%	.16
Human-supported	20	−0.354	−0.596 to −0.112	78.30%	*.006*
Comparator					
Inert control	15	−0.178	−0.307 to −0.050	5.80%	*.01*
Enhanced control	20	−0.322	−0.592 to −0.053	83.00%	*.02*
Duration					
Short-term	16	−0.456	−0.782 to −0.131	82.10%	.*009*
Long-term	19	−0.13	−0.254 to −0.006	35.10%	.*04*
Outcome hierarchy					
Primary	19	−0.215	−0.428 to −0.002	63.50%	.*049*
Secondary	16	−0.316	−0.582 to −0.049	78.10%	.*02*
Mentoring					
Passive tracking	12	−0.224	−0.507 to 0.060	84.00%	.11
Active monitoring	23	−0.286	−0.498 to −0.075	62.80%	.*01*

a Italicized values indicate statistically significant results (*P*<.05).

b SMD: standardized mean difference.

##### Significant and Borderline Moderators

Intervention duration was identified as a significant moderator of efficacy (*Q*=3.98, *P*=.05). Short-term interventions (≤8 weeks) demonstrated a significant and more pronounced reduction in caregiver burden (SMD −0.456, 95% CI −0.782 to −0.131; *P*=.009; τ²=0.276, *τ*=0.526), whereas long-term interventions showed a smaller, though still significant, effect (SMD −0.130, 95% CI −0.254 to −0.006; *P=.*04; τ²=0.044, *τ*=0.209). Furthermore, Delivery Modality showed a strong moderating trend (*Q*=4.71, *P*=.10). Mobile-based interventions (Apps/IM) yielded the largest pooled effect size (SMD −0.505, 95% CI −0.914 to −0.097; *P*=.02; τ²=0.303, *τ*=0.551), followed by Web-based platforms (SMD −0.183, 95% CI −0.410 to 0.043; *P=.*10; τ²=0.106, *τ*=0.326), while Video/Teleconsultation (VC) showed the smallest and nonsignificant effect (SMD −0.086, 95% CI −0.240 to 0.069; *P*=.24; τ²=0.037, *τ*=0.193). Notably, VC-based subgroups exhibited zero within-group heterogeneity (*I*^2^=0%), suggesting a highly consistent, albeit modest, effect across diverse settings.

##### Impact of Guidance and Monitoring Strategies

Regarding guidance and monitoring, neither Guidance Category (*P*=.16) nor Clinical Monitoring (*P*=.70) reached statistical significance for between-group differences; however, clear patterns of within-group efficacy emerged. Interventions incorporating Human-Supported components (SMD −0.354, 95% CI −0.596 to −0.112; *P=.*006; τ²=0.203, *τ*=0.450) and Active Monitoring (SMD −0.286, 95% CI −0.498 to −0.075; *P=.*01; τ²=0.172, *τ*=0.415) both demonstrated significant burden reduction, whereas Self-guided programs (SMD −0.143, 95% CI −0.348 to 0.062; *P=.*16; τ²=0.097, *τ*=0.311) and Passive Tracking (SMD −0.224, 95% CI −0.507 to 0.060; *P*=.11; τ²=0.162, *τ*=0.402) failed to achieve statistical significance. Similarly, both forms of Interaction yielded significant results, with Asynchronous delivery (SMD −0.292, 95% CI −0.544 to −0.040; *P*=.03; τ²=0.217, *τ*=0.466) remaining more robustly supported than Synchronous delivery (SMD −0.224, 95% CI −0.429 to −0.018; *P*=.04; τ²=0.105, *τ*=0.324). Analysis of Comparator type revealed that eHealth interventions were effective regardless of whether they were compared against Inert Controls (SMD −0.178, 95% CI −0.307 to −0.050; *P*=.01; *I*^2^=5.8%; τ²=0.042, *τ*=0.204) or Enhanced Controls (SMD −0.322, 95% CI −0.592 to −0.053; *P*=.02; τ²=0.258, *τ*=0.508). The remarkably low heterogeneity in the Inert Control subgroup (*I*^2^=5.8%).

##### Caregiver and Outcome Characteristics

No significant differences were observed across Caregiver Dominant Types (*P*=.67) or Outcome Hierarchy (*P*=.53). Analysis of caregiver profiles revealed that interventions targeting Spousal-dominant groups yielded a remarkably consistent and significant effect (*k*=10, SMD −0.183, 95% CI −0.295 to −0.072; *P*=.005; τ²=0.013, *τ*=0.113) with zero heterogeneity (*I^2^*=0%), whereas Adult-child (SMD −0.284, 95% CI −0.583 to 0.015; *P*=.06; τ²=0.237, *τ*=0.487) and Mixed (SMD −0.305, 95% CI −0.720 to 0.110; *P*=.13; τ²=0.248, *τ*=0.498) groups exhibited high variability (*I^2^*>74%). Finally, eHealth interventions were effective regardless of whether burden was assessed as a Primary (SMD −0.215, 95% CI −0.428 to −0.002; *P*=.049; τ²=0.153, *τ*=0.391) or Secondary (SMD −0.316, 95% CI −0.582 to −0.049; *P=.*02; τ²=0.182, *τ*=0.427) outcome.

### Synthesis of Secondary Outcome: Depressive Symptoms

#### Overall Effect and PI

**Figure 4.**
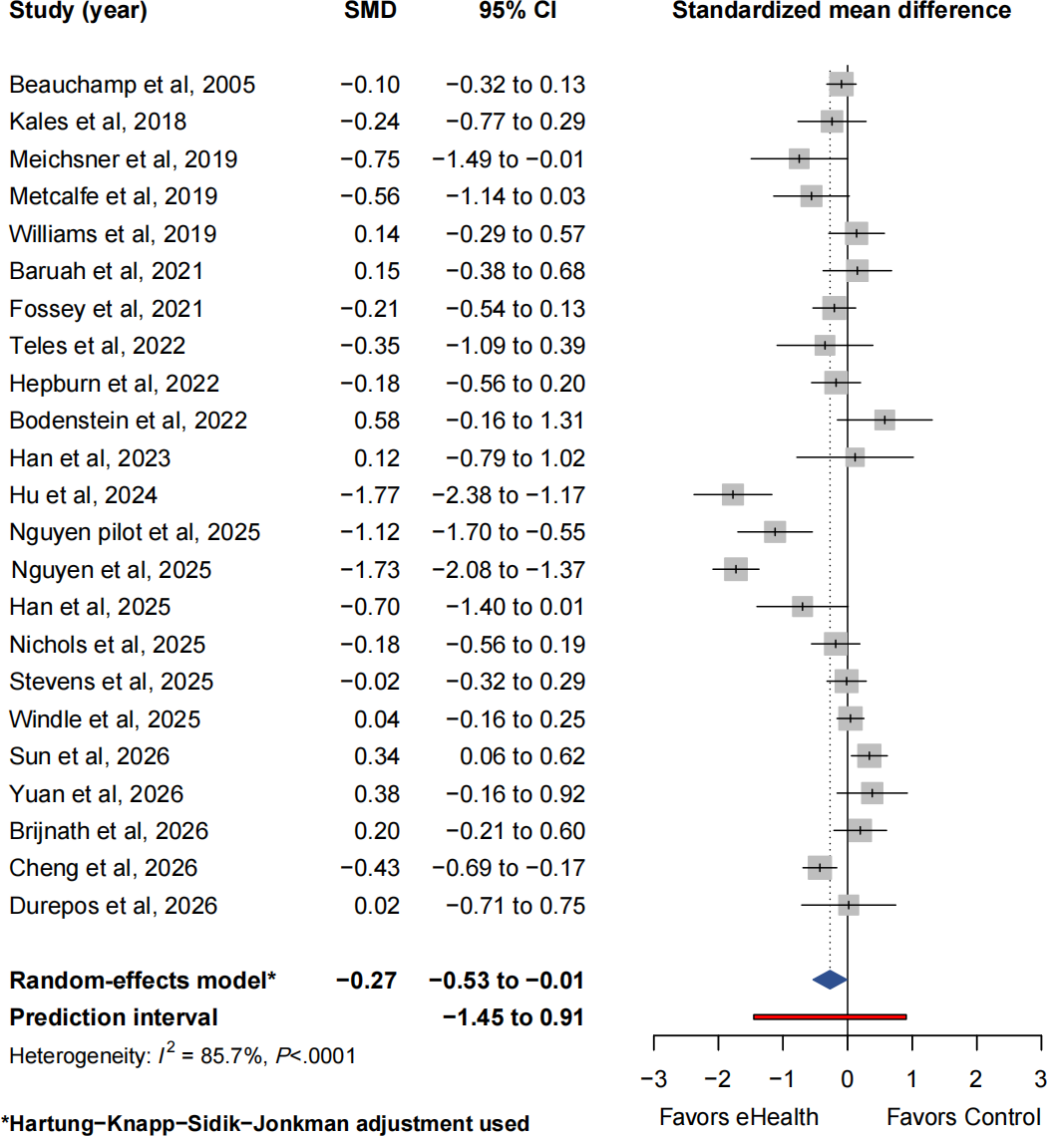
Forest plot of depressive symptoms, showing Hartung-Knapp-Sidik-Jonkman–adjusted random-effects estimates and 95% prediction intervals [27,31-34,36-41,44,50-54,56-61]. SMD: standardized mean difference.

Twenty-three studies involving 2467 participants (*k*=23; intervention: n=1261; control: n=1206) reported depressive symptoms among caregivers. Meta-analysis using a random-effects model with the HKSJ adjustment indicated a statistically significant reduction in depressive symptoms associated with eHealth interventions (SMD −0.27, 95% CI −0.53 to −0.01; *P*=.04; τ^2^=0.31, *τ*=0.55; 95% PI −1.45 to 0.91; Figure 4 [27,31-34,36-41,44,50-54,56-61]). Substantial heterogeneity was observed across studies (*I²*=85.7%, 95% CI 79.8%-89.9%; *Q*=154.07, *df*=22; *P*<.001).

#### Subgroup Analyses for Depressive Symptoms

Prespecified subgroup analyses were conducted to investigate the sources of substantial heterogeneity observed in the secondary outcome (Table 3 and Figure S2 in Multimedia Appendix 1 [27,31-34,36-41,44,50-54,56-61]).

**Table 3.** Subgroup analyses of eHealth interventions on depressive symptoms (*k*=23).

Variables and levels	*K*	SMD^a^	95% CI	*I* ^2^	*P*_within_
Caregiver dominant type					
Adult-child-dominant	12	−0.214	−0.623 to 0.194	90.40%	.27
Spousal-dominant	6	−0.234	−0.532 to 0.063	16.10%	.10
Mixed	5	−0.42	−1.499 to 0.658	88.00%	.34
Modality					
Web-based	10	−0.15	−0.347 to 0.047	47.30%	.12
App/IM (Mobile)	7	−0.611	−1.461 to 0.239	94.80%	.13
Video/Tele (VC)	6	−0.019	−0.433 to 0.394	32.00%	.91
Interaction					
Asynchronous	14	−0.267	−0.612 to 0.078	88.70%	.12
Synchronous	9	−0.281	−0.786 to 0.224	78.30%	.24
Guidance category					
Self-guided	10	−0.064	−0.322 to 0.193	67.20%	.59
Human-supported	13	−0.423	−0.859 to 0.013	88.70%	.06
Comparator					
Inert control	10	−0.071	−0.359 to 0.218	69.30%	.59
Enhanced control	13	−0.432	−0.852 to −0.012	89.70%	*.045* ^b^
Duration					
Short-term	11	−0.504	−1.034 to 0.027	90.40%	.06
Long-term	12	−0.052	−0.229 to 0.125	54.40%	.53
Outcome hierarchy					
Secondary	15	−0.225	−0.536 to 0.087	76.90%	.14
Primary	8	−0.357	−0.945 to 0.230	92.10%	.19
Monitoring					
Passive tracking	11	−0.187	−0.591 to 0.217	90.50%	.33
Active monitoring	12	−0.361	−0.757 to 0.035	75.80%	.07

a SMD: standardized mean difference.

b Italicized value indicates statistically significant result (*P*<.05).

##### Key Drivers and Borderline Moderators

Although no single variable reached the traditional threshold for subgroup differences, Intervention Duration emerged as a key moderator with a strong trend toward significance (*P*=.07). Short-term interventions (≤8 weeks) demonstrated a larger potential effect size (SMD −0.504, 95% CI −1.034 to 0.027; *P*=.06; τ²=0.541, *τ*=0.735) than Long-term programs (SMD −0.052, 95% CI −0.229 to 0.125; *P*=.53; τ²=0.051, *τ*=0.225). Notably, the Comparator Type and Guidance Category also exhibited meaningful patterns. Interventions showed significant within-group efficacy when compared against Enhanced Controls (SMD −0.432, 95% CI −0.852 to −0.012; *P*=.045; τ²=0.412, *τ*=0.642), whereas comparisons with Inert Controls were nonsignificant (SMD −0.071, 95% CI −0.359 to 0.218; *P*=.59; τ²=0.126, *τ*=0.355). Similarly, Human-Supported guidance (SMD −0.423, 95% CI −0.859 to 0.013; *P*=.06; τ²=0.448, *τ*=0.669) showed a stronger trend toward within-group efficacy than Self-guided programs (SMD −0.064, 95% CI −0.322 to 0.193; *P*=.59; τ²=0.096, *τ*=0.310).

##### Platform and Technical Implementation

Analysis of Modality (*P*=.30) and Interaction Type (*P*=.96) confirmed that the digital delivery format did not significantly alter intervention outcomes. While Mobile-based apps/IM (SMD −0.611, 95% CI −1.461 to 0.239; *P*=.13; τ²=0.781, *τ*=0.884) showed the largest point estimate among platforms, high within-group heterogeneity (*I*^2^=94.8%) resulted in wide CIs (Web-based: SMD −0.150, 95% CI −0.347 to 0.047; *P*=.12; τ²=0.054, *τ*=0.232; Video/Tele (VC): SMD −0.019, 95% CI −0.433 to 0.394; *P*=.91; τ²=0.095, *τ*=0.308). Furthermore, Active Monitoring (eg, real-time tracking with feedback) showed a stronger trend toward reduction (SMD −0.361, 95% CI −0.757 to 0.035; *P*=.07; τ²=0.314, *τ*=0.560) than Passive Tracking (SMD −0.187, 95% CI −0.591 to 0.217; *P*=.33; τ²=0.316, *τ*=0.563).

##### Caregiver Characteristics and Generalizability

The efficacy of eHealth interventions appeared remarkably consistent across different caregiver demographics. No significant differences were found for Caregiver Dominant Type (*P*=.89), with Adult-child (SMD −0.214, 95% CI −0.623 to 0.194; *P*=.27; τ²=0.350, *τ*=0.592), Spousal (SMD −0.234, 95% CI −0.532 to 0.063; *P*=.10; τ²=0.048, *τ*=0.218), and Mixed (SMD −0.420, 95% CI −1.499 to 0.658; *P*=.34; τ²=0.667, *τ*=0.817) caregiver groups all showing similar directions of effect. Of note, the Spousal-dominant subgroup exhibited the highest level of evidence consistency (*I*^2^=16.1%). Similarly, no meaningful differences were observed across interaction formats, with comparable effects for Asynchronous (SMD −0.267, 95% CI −0.612 to 0.078; *P*=.12; τ²=0.304, *τ*=0.551) and Synchronous interventions (SMD −0.281, 95% CI −0.786 to 0.224; *P*=.24; τ²=0.356, *τ*=0.596). Finally, the impact remained stable regardless of whether depressive symptoms were evaluated as a Primary (SMD −0.357, 95% CI −0.945 to 0.230; *P*=.19; τ²=0.430, *τ*=0.656) or Secondary (SMD −0.225, 95% CI −0.536 to 0.087; *P*=.14; τ²=0.260, *τ*=0.510) outcome (*P*=.65).

### Exploration of Heterogeneity: Meta-Regression

Univariate and multivariate meta-regression analyses were conducted examining caregiver age, proportion of female participants, and attrition rates (Table 4).

**Figure 5.**
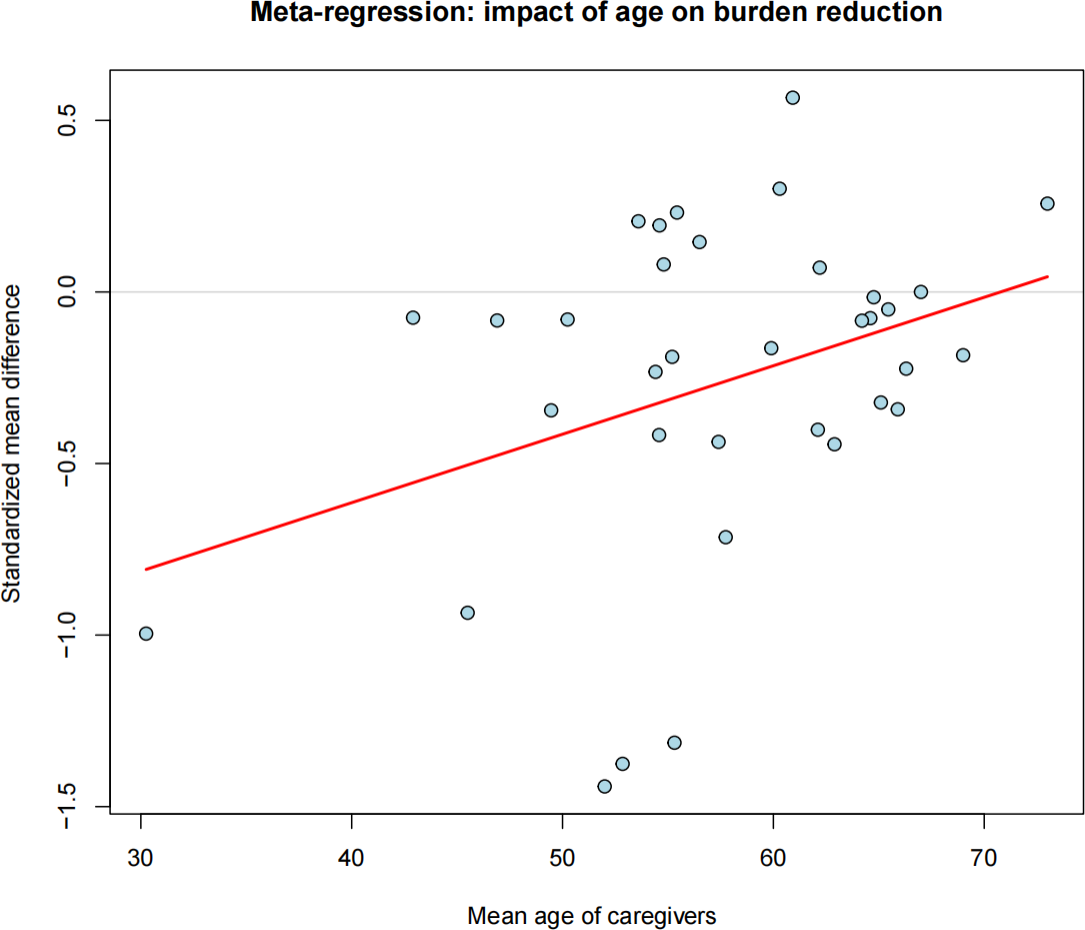
Meta-regression bubble plot of mean age of caregivers on the intervention effect (standardized mean difference). The red line indicates the fitted meta-regression trend line.

For the primary outcome (Caregiver Burden; *k*=35), caregiver age emerged as a significant moderator (β=.02, 95% CI 0.0008 to 0.0392; *Z*=2.044, *P*=.049; Figure 5). In contrast, neither the proportion of female participants (*P*=.44) nor the study attrition rate (*P*=.36) significantly influenced the treatment effect. In the multivariate model, age showed borderline significance (β=.0209, 95% CI −0.0009 to 0.0427; *P*=.07), and the intercept remained significant (β=−1.542, 95% CI −2.7495 to −0.3347; *P*=.02).

**Table 4.** Meta-regression analysis of potential moderators for eHealth intervention efficacy.

Moderator	Coefficient, β	SE	95% CI	*Z* value	*P* value
Caregiver burden (*k*^a^=35)					
Age (years), mean	0.0200	0.0098	0.0008 to 0.0392	2.044	*.049* ^b^
Female, %	0.0046	0.0059	−0.0070 to 0.0162	0.788	.44
Attrition, %	0.0043	0.0047	−0.0049 to 0.0135	0.922	.36
Multivariate model					
Intercept	−1.5421	0.616	−2.7495 to −0.3347	−2.504	.02
Age (years)	0.0209	0.0111	−0.0009 to 0.0427	1.877	.07
Female, %	−0.0001	0.0064	−0.0126 to 0.0124	−0.023	.98
Attrition rate	0.0050	0.0045	−0.0038 to 0.0138	1.100	.28
Depressive symptoms (*k*=23)					
Age (years), mean	0.0162	0.0184	−0.0199 to 0.0523	0.877	.39
Female, %	0.0023	0.0104	−0.0181 to 0.0227	0.222	.83
Attrition, %	0.0087	0.0068	−0.0046 to 0.0220	1.276	.22
Multivariate model					
Intercept	−1.4264	1.1837	−3.7464 to 0.8936	−1.205	.24
Age (years)	0.0149	0.0199	−0.0241 to 0.0539	0.748	.46
Female, %	0.0018	0.0112	−0.0201 to 0.0237	0.161	.87
Attrition rate	0.0089	0.0072	−0.0052 to 0.0230	1.237	.23

a *k*: number of studies.

b Italicized value indicates statistically significant result (*P*<.05).

For the secondary outcome (Depressive Symptoms) (*k*=23), the meta-regression results were consistent with the burden analysis in terms of direction but did not reach statistical significance. The intervention effect on depression was not significantly moderated by caregiver age (β=.0162, 95% CI −0.0199 to 0.0523; *P*=.39), female percentage (*P*=.83), or attrition rate (*P*=.22). The multivariate model confirmed that these factors collectively did not explain the substantial residual heterogeneity.

### Small-Study Effects and Publication Bias

**Figure 6.**
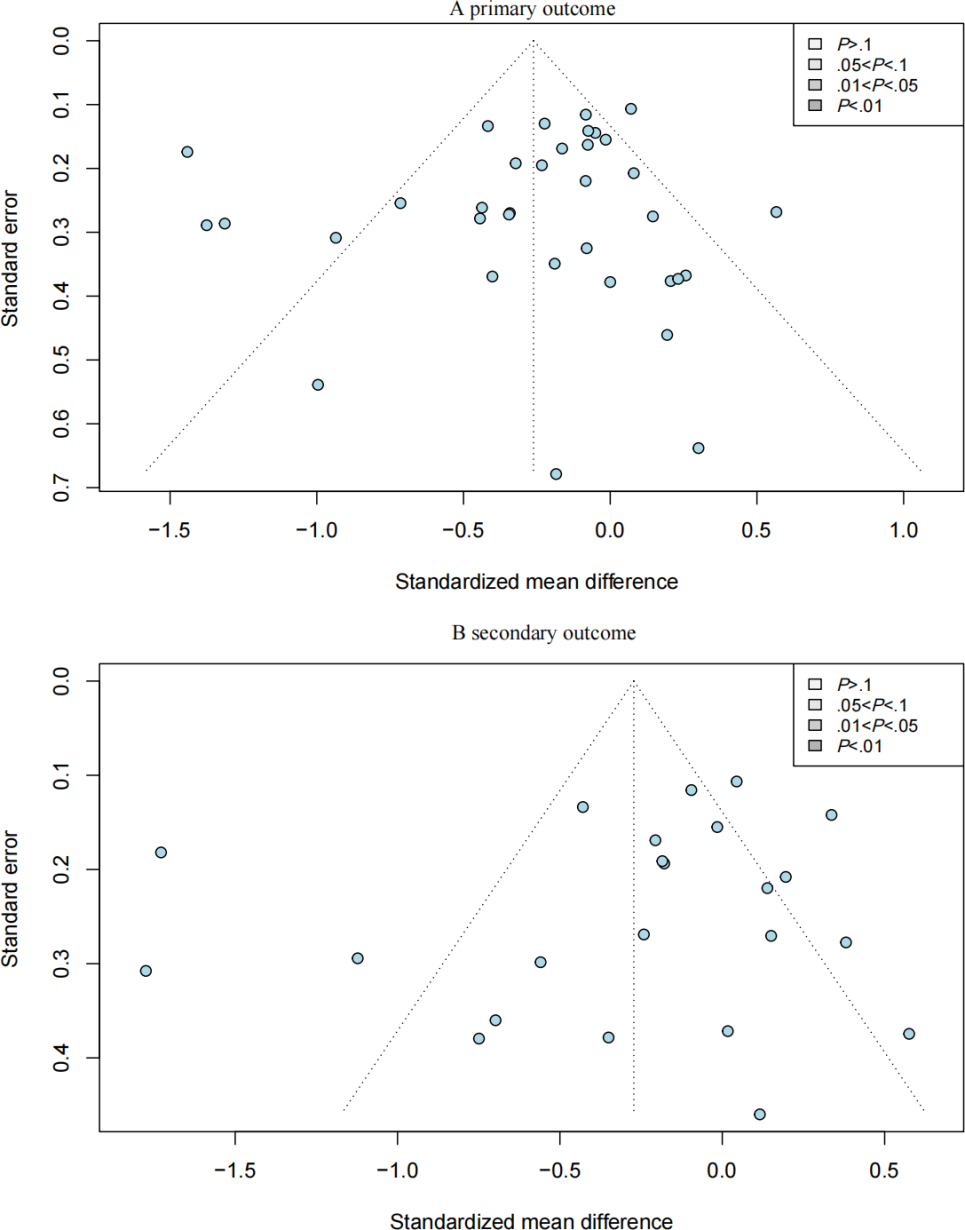
Contour-enhanced funnel plot for assessment of publication bias. (A) primary outcome (caregiver burden) and (B) secondary outcome (depressive symptoms). The plots indicate symmetric distributions across both outcomes.

Egger tests indicated no significant funnel plot asymmetry for caregiver burden (*t*_33_=−0.808; *P*=.42) or depressive symptoms (*t*_21_=−0.954; *P*=.35). Contour-enhanced funnel plots (Figure 6A for caregiver burden and Figure 6B for depressive symptoms) confirmed symmetric distributions, suggesting that observed heterogeneity is attributable to study characteristics rather than publication bias.

### Sensitivity Analysis and Robustness

Sensitivity analyses were conducted by focusing on 27 high-quality studies (N=2885) for the primary outcome and 19 studies (N=2059) for the secondary outcome, after excluding those identified with a high risk of bias. For the primary outcome (Caregiver Burden), the pooled effect for caregiver burden remained statistically significant and increased in magnitude (SMD −0.31, 95% CI −0.50 to −0.13; *t*_26_=−3.47; *P*=.002; 95% PI −1.28 to 0.59; τ²=0.169, *τ*=0.412) (Table S3 in Multimedia Appendix 1). Heterogeneity remained substantial (*I^2^*=77.0%; *Q*=113.19; *P*<.001). This PI indicates that while the average effect is beneficial, the outcome in a specific clinical setting could vary.

Regarding the secondary outcome (Depressive Symptoms), the pooled effect yielded a borderline significant reduction (SMD −0.31, 95% CI −0.63 to 0.01; *t*_18_=−2.06; *P*=.05; 95% PI −1.63 to 1.01; τ²=0.371, *τ*=0.609). While heterogeneity remained high (*I*²=88.1%, *P*<.001), the 95% PI and the borderline significant *P* value support that eHealth interventions provide a nonsignificant trend for depressive symptoms. Collectively, these results derived from the conservative HKSJ model confirmed that the observed clinical benefits of eHealth interventions are not driven by low-quality studies and remain robust even under the most rigorous inclusion criteria.

### Quality of Evidence (GRADE)

Using the GRADE approach, the certainty for both caregiver burden and depressive symptoms was rated as moderate (Table 5). Evidence was downgraded 1 level due to risk of bias associated with lack of participant blinding and reliance on self-reported outcomes. Despite high heterogeneity, consistent effect direction across studies supported a moderate rating.

**Table 5.** Summary of findings and Grading of Recommendations, Assessment, Development, and Evaluation certainty of evidence.

Certainty assessment							Patients, n		Effect (95% CI)		Certainty	Importance
Studies, N	Study design	Risk of bias	Inconsistency	Indirectness	Imprecision	Other considerations	eHealth interventions	Normal care	Relative	Absolute		
Caregiver burden												
35	Randomized trials	Serious^a^	Not serious	Not serious	Not serious	None	1700	1688	N/A^b^	SMD^c^ −0.26 SD lower (−0.42 lower to −0.10 lower)	⨁⨁⨁○^d^ Moderate^a^	Critical
Depressive symptoms												
23	Randomized trials	Serious^a^	Not serious	Not serious	Not serious	None	1261	1206	N/A^b^	SMD −0.27 SD lower (−0.53 lower to 0.01 lower)	⨁⨁⨁○^d^ Moderate^a^	Critical

a Downgraded 1 level due to lack of blinding (D2) and subjective self-reporting (D4) in most randomized controlled trials.

b Not applicable.

c SMD: standardized mean difference.

d ⨁ indicates level of evidence; ○ indicates no downgrade in certainty within the GRADE (Grading of Recommendations, Assessment, Development, and Evaluation) framework.

## Discussion

### Principal Findings

This systematic review and meta-analysis of 35 RCTs suggests that eHealth interventions are associated with a small but statistically significant reduction in caregiver burden and depressive symptoms among informal caregivers of people with dementia. Although pooled estimates favor eHealth interventions, the 95% PI indicates that the effect in a future specific clinical context could range from a substantial benefit to no effect. This distinction suggests that while eHealth interventions are validated at the population level, their effectiveness in real-world settings is highly context-dependent. The moderate certainty of this evidence, assessed by GRADE, reflects consistent effects direction alongside recognized limitations of psychosocial trials, particularly the inability to blind participants and outcome assessors.

A key observation is that eHealth interventions incorporating human support or active monitoring tend to yield larger effect sizes than fully self-guided approaches. Although subgroup analyses were not statistically significant, consistent within-group patterns suggest that human involvement (eg, professional feedback, coaching, or monitoring) may enhance engagement and adherence [62,63]. The magnitude of effect observed in this review exceeds the negligible effects reported in some prior meta-analyses (eg, SMD≈−0.06) [19], yet remains smaller than those reported for structured, education-based interventions (eg, *g*=−0.45) [2], and is consistent with recent syntheses of home-based digital interventions reporting small-to-moderate but heterogeneous effects [64]. The wider literature, however, remains inconsistent, with some studies reporting moderate short-term benefits and others demonstrating small or nonsignificant effects, particularly for caregiver burden and depressive outcomes [17-19,21]. This heterogeneity likely reflects variation in intervention intensity, degree of human support, and methodological differences across trials, including attrition, outcome measurement, and duration of follow-up. Importantly, while confidence intervals represent the average effect, the wide PIs underscore substantial variability in outcomes across contexts. Collectively, these findings indicate that eHealth interventions yield modest and heterogeneous effects, driven more by intervention characteristics than by uniformly strong efficacy.

Subgroup analyses further suggest that short-term (≤8 weeks) and mobile-based interventions tend to demonstrate relatively stronger effects on caregiver burden reduction. While consistent with prior evidence that time-limited digital interventions can improve proximal psychosocial outcomes, effects on caregiver burden remain inconsistent [19]. Compared with earlier reports of larger effects (eg, *d*=−0.65) [17], and more recent estimates showing smaller effects (eg, SMD=−0.21) [18], the pooled estimates in this review are comparatively conservative, potentially reflecting methodological differences, including the use of the HKSJ adjustment. Although shorter-duration (≤2 months) and mobile- or web-based interventions appeared more likely to yield statistically significant reductions in caregiver burden, these findings were not robust under substantial heterogeneity (*I*²≈75%‐92%) and variations in study design [17,18,65]. Overall, effects on caregiver burden remain small or nonsignificant, particularly for long-term outcomes and quality of life. One plausible explanation is that short-term or mobile-based interventions primarily influence proximal outcomes, while failing to produce sustained changes in more complex outcomes such as caregiver burden and quality of life, potentially due to limited intensity or insufficient personalization [66]. Taken together, these findings suggest that intervention effectiveness is determined more by design features, outcome selection, and intensity than by delivery modality alone [16,67].

Meta-regression analyses demonstrated a potential moderating effect of caregiver age, with reduced effectiveness observed in older populations in univariate models. However, these findings suggest that intervention effectiveness is determined more by design features, outcome selection, and intensity than by delivery modality alone. This pattern aligns with existing evidence suggesting that digital interventions for caregivers generally yield small or nonsignificant effects and limited improvements in quality of life [66], and that benefits may not be uniformly distributed across subgroups. Age-related differences may be attributable to broader digital divide factors, including variability in digital literacy, usability challenges, and technology-related self-efficacy [68]. Evidence from gerontechnology and digital health research indicates that adoption and sustained engagement are strongly influenced by usability and contextual fit, with digital literacy demands and cognitive load representing persistent barriers [69,70]. In addition, broader syntheses of digital therapeutics adoption identify mistrust, usability constraints, and insufficiently tailored onboarding processes as key barriers among older populations [71]. Accordingly, differential effectiveness by age is more plausibly explained by variation in engagement and usability rather than by inherent differences in treatment response.

Overall, the findings of this review are consistent with recent umbrella and meta-analytic evidence suggesting that intervention heterogeneity, rather than delivery modality per se, is the principal driver of variability in effect estimates across studies [17,18,21]. This reinforces the interpretation that effectiveness is not determined solely by the presence of digital components but by how interventions are designed, implemented, and aligned with user needs. Accordingly, standardized, one-size-fits-all digital approaches are unlikely to achieve optimal outcomes. Future interventions should instead prioritize adaptability and personalization, alongside the deliberate integration of human support elements (eg, guidance, feedback, or monitoring), to enhance user engagement, adherence, and overall effectiveness.

### Limitations

Several limitations should be considered when interpreting these findings. First, the wide PI indicates substantial between-study variability, suggesting that intervention effects may differ considerably across real-world settings. Second, as common in psychosocial and digital health research, most studies were rated as having “some concerns” or “high risk” of bias, primarily due to the inability to blind participants (RoB 2 Domain 2) and reliance on self-reported outcomes (Domain 4). Third, high attrition rates in several pilot trials may have affected internal validity [35,38], although meta-regression did not identify attrition as a significant moderator. Fourth, while subgroup analyses identified potentially important moderators (eg, intervention duration, delivery modality, and level of human support), these findings should be interpreted cautiously due to limited statistical power and persistent heterogeneity. Furthermore, although the screening process was rigorous, the preliminary title-based triage may have introduced a minimal risk of inadvertently excluding relevant studies. Finally, this review was restricted to English-language peer-reviewed publications, potentially excluding relevant gray literature or region-specific implementations.

### Innovation, Contributions, and Real-World Implications

This review extends the existing literature in several important ways. Methodologically, the application of the HKSJ approach, alongside the reporting of PIs, provides more conservative and clinically informative estimates of intervention effects. Analytically, it moves beyond evaluating overall effectiveness by exploring potential moderators, offering insights into the conditions under which eHealth interventions may be more effective. Although subgroup differences were not statistically significant, the observed patterns highlight the potential importance of intervention characteristics such as human support and duration [10].

From a practical perspective, these findings have important implications for clinical practice, digital health development, and health policy. Clinically, guided eHealth interventions could be integrated into dementia care pathways as scalable support tools, particularly during high-stress periods. For digital health developers, platforms should be grounded in evidence-based psychological frameworks [17], and our results underscore the potential value of embedding human-supported components, such as coaching, feedback, or monitoring, within digital platforms, rather than relying on fully self-guided designs. At the health system level, the evidence points toward the potential of a hybrid care model in which digital infrastructure is complemented by training human facilitators or “digital coaches,” whose involvement appears linked to intervention effectiveness [62,63]. Usability and embedded support appear more critical than baseline digital literacy for successful adoption [71].

### Conclusions

This review provides timely evidence that eHealth interventions are associated with modest reductions in caregiver burden and improvements in depressive symptoms among informal caregivers of people with dementia. By applying the HKSJ framework with PIs, this study offers more robust and practice-relevant estimates than prior reviews. Interpretation of these findings requires integrating multiple dimensions of evidence, as statistically significant average effects based on 95% CIs coexist with wide 95% PIs, indicating substantial heterogeneity and suggesting that real-world effectiveness may range from meaningful benefit to minimal impact. This variability, together with the identified risk of bias, particularly the lack of participant blinding, and an overall moderate level of certainty based on GRADE, indicates that intervention effectiveness is context-dependent and influenced by both methodological and implementation factors. Subgroup analyses indicate potential benefits of human-supported and shorter-duration interventions, although substantial heterogeneity persists. Future research should focus on optimizing hybrid digital-human intervention models, improving accessibility across diverse populations, and generating high-quality evidence with longer follow-up to enhance consistency and real-world applicability.

### What Is Already Known

These include the following:

- Informal caregivers of people with dementia experience high levels of psychological burden; eHealth interventions offer a promising scalable approach for caregiver support.

- Previous meta-analyses often relied on conventional statistical models, which may underestimate uncertainty and yield overly narrow CIs in the presence of substantial heterogeneity.

### What This Paper Adds

These include the following:

- Methodological rigor: This review applies PRISMA-S reporting standards and uses the HKSJ method and PIs, providing a more conservative and practice-relevant estimate of intervention effectiveness.
- Intervention characteristics insights: Interventions incorporating human support and mobile-based delivery were associated with more consistent effects, whereas fully self-guided approaches showed less consistent evidence of benefit.
- Demographic moderators: Meta-regression reveals that caregiver age appears to influence intervention effectiveness, with reduced effects observed in older populations, highlighting the importance of age-sensitive intervention design.

## Supplementary material

10.2196/78568 Multimedia Appendix 1 Supplementary tables and figures including search strategy (Table S1), characteristics of included randomized controlled trials of eHealth interventions for informal caregivers of people with dementia (Table S2), comparison of primary and sensitivity meta-analyses (Table S3), and forest plots of subgroup analyses for caregiver burden and depressive symptoms (Figures S1 and S2).

10.2196/78568 Checklist 1 PRISMA 2020 expanded checklist.

10.2196/78568 Checklist 2 PRISMA-S checklist.

10.2196/78568 Checklist 3 PRISMA 2020 for abstracts checklist.

## Abbreviations

CENTRAL: Cochrane Central Register of Controlled Trials
GRADE: Grading of Recommendations, Assessment, Development, and Evaluation
HKSJ: Hartung-Knapp-Sidik-Jonkman
IM: Instant Messaging
MeSH: Medical Subject Headings
PI: prediction interval
PICOS: Population, Intervention, Comparison, Outcomes, and Study Design
PRISMA: Preferred Reporting Items for Systematic Reviews and Meta-Analyses
PRISMA-S: Preferred Reporting Items for Systematic Reviews and Meta-Analyses—Search extension
RCT: randomized controlled trial
RoB 2: Cochrane Risk of Bias 2
SMD: standardized mean difference
VC: videoconferencing
