# Supplementary material for: Effectiveness of eHealth Interventions in Alleviating Burden on Informal Caregivers of People With Dementia: Systematic Review and Meta-Analysis of Randomized Controlled Trials
Source: J Med Internet Res. 2026 Jun 3;28:e78568. doi: 10.2196/78568 (PMC13234497; doi:10.2196/78568)
Supplement: Multimedia Appendix 1 [file jmir-v28-e78568-s001.docx]

Table S1. Search strategy.

| Database | Term | Search Strategy | Result |
| --- | --- | --- | --- |
| PubMed | #1 Caregiver | "Caregivers"[MeSH] OR caregiver*[Title/Abstract] OR carer*[Title/Abstract] OR "family caregiver*"[Title/Abstract] OR "informal caregiver*"[Title/Abstract] OR "spouse caregiver*"[Title/Abstract] OR "adult child caregiver*"[Title/Abstract] OR "unpaid caregiver*"[Title/Abstract] OR "primary caregiver*"[Title/Abstract] | 154,670 |
|  | #2 Dementia | "Dementia"[MeSH] OR "Alzheimer Disease"[MeSH] OR dementia*[Title/Abstract] OR alzheimer*[Title/Abstract] OR "cognitive impairment"[Title/Abstract] OR "major neurocognitive disorder"[Title/Abstract] | 439,906 |
|  | #3 eHealth | "Telemedicine"[MeSH] OR "Mobile Applications"[MeSH] OR eHealth[Title/Abstract] OR e-health[Title/Abstract] OR mHealth[Title/Abstract] OR m-health[Title/Abstract] OR telehealth[Title/Abstract] OR telemedicine[Title/Abstract] OR "mobile health"[Title/Abstract] OR "digital health"[Title/Abstract]OR "web-based"[Title/Abstract] OR "internet-based"[Title/Abstract] OR "online program*"[Title/Abstract] OR "digital program*"[Title/Abstract] OR "online intervention*"[Title/Abstract] OR "digital intervention*"[Title/Abstract] OR "internet-delivered"[Title/Abstract] OR "mobile app*"[Title/Abstract] OR smartphone*[Title/Abstract] OR "virtual support"[Title/Abstract] OR "remote support"[Title/Abstract] | 211,552 |
|  | #4 | #1 AND #2 AND #3 | 1,159 |
| EMbase (via Elsevier) | #1 Caregiver | caregiver'/exp OR caregiver* OR carer* OR 'family caregiver*' OR 'informal caregiver*' OR 'spouse caregiver*' OR 'adult child caregiver*' OR 'unpaid caregiver*' OR 'primary caregiver*' | 246,235 |
|  | #2 Dementia | dementia' /exp OR 'alzheimer disease' OR dementia* OR alzheimer* OR 'cognitive impairment' OR 'major neurocognitive disorder' | 695,858 |
|  | #3 eHealth | telemedicine' /exp OR 'mobile applications' OR 'remote consultation' OR ehealth OR 'e health' OR mhealth OR 'm health' OR telehealth OR telemedicine OR 'mobile health' OR 'digital health' OR 'web-based' OR 'internet-based' OR 'online program*' OR 'digital program*' OR 'online intervention*' OR 'digital intervention*' OR 'internet-delivered' OR 'mobile app*' OR smartphone* OR 'virtual support' OR 'remote support' | 324,738 |
|  | #4 | #1 AND #2 AND #3 | 2,031 |
| Web of Science Core Collection (WoS CC) | #1 Caregiver | TS=(caregiver* OR carer* OR "family caregiver*" OR "informal caregiver*" OR "spouse caregiver*" OR "adult child caregiver*" OR "unpaid caregiver*" OR "primary caregiver*") | 167,296 |
|  | #2 Dementia | TS=(Dementia OR "Alzheimer Disease" OR alzheimer* OR "cognitive impairment" OR "major neurocognitive disorder") | 548,240 |
|  | #3 eHealth | TS=(Telemedicine OR "Mobile Applications" OR "Remote Consultation" OR eHealth OR "e-health" OR mHealth OR "m-health" OR telehealth OR "mobile health" OR "digital health" OR "web-based" OR "internet-based" OR "online program*" OR "digital program*" OR "online intervention*" OR "digital intervention*" OR "internet-delivered" OR "mobile app*" OR smartphone* OR "virtual support" OR "remote support") | 353,003 |
|  | #4 | #1 AND #2 AND #3 | 1,305 |
| Cochrane Central Register of Controlled Trials (CENTRAL) | #1 Caregiver | caregiver* OR carer* OR (family NEXT caregiver*) OR (informal NEXT caregiver*) OR ("Spouse" NEXT caregiver*) OR (adult NEXT child NEXT caregiver*) OR (unpaid NEXT caregiver*) OR (primary NEXT caregiver*) | 30,943 |
|  | #2 Dementia | dementia OR "Alzheimer Disease" OR dementia* OR alzheimer* OR ("cognitive NEXT impairment") OR ("major NEXT neurocognitive NEXT disorder") | 39,169 |
|  | #3 eHealth | telemedicine OR (Mobile NEXT Applications) OR (Remote NEXT Consultation) OR eHealth OR e-health OR mHealth OR m-health OR telehealth OR telemedicine OR (mobile NEXT health) OR (digital NEXT health) OR "web-based" OR "internet-based" OR (online NEXT program*) OR (digital NEXT program*) OR (online NEXT intervention*) OR (digital NEXT intervention*) OR ("internet-delivered") OR (mobile NEXT app*) OR smartphone* OR (virtual NEXT support) OR (remote NEXT support) | 50,605 |
|  | #4 | #1 AND #2 AND #3 | 589 |
| Scopus | #1 Caregiver | TITLE-ABS-KEY (caregiver* OR carer* OR "family caregiver" OR "informal caregiver" OR "spouse caregiver" OR "adult child caregiver" OR "unpaid caregiver" OR "primary caregiver") | 229,277 |
|  | #2 Dementia | TITLE-ABS-KEY (Dementia OR "Alzheimer Disease" OR alzheimer* OR "cognitive impairment" OR "major neurocognitive disorder") | 593,057 |
|  | #3 eHealth | TITLE-ABS-KEY (Telemedicine OR "Mobile Applications" OR "Remote Consultation" OR eHealth OR "e-health" OR mHealth OR "m-health" OR telehealth OR "mobile health" OR "digital health" OR "web-based" OR "internet-based" OR "online program*" OR "digital program*" OR "online intervention*" OR "digital intervention*" OR "internet-delivered" OR "mobile app*" OR smartphone* OR "virtual support" OR "remote support") | 537,759 |
|  | #4 | TITLE-ABS-KEY((caregiver OR carer OR "family caregiver" OR "informal caregiver" OR "spouse caregiver" OR "adult child caregiver" OR "unpaid caregiver" OR "primary caregiver") AND (dementia OR alzheimer* OR "cognitive impairment" OR "major neurocognitive disorder") AND (Telemedicine OR "Mobile Applications" OR "Remote Consultation" OR eHealth OR "e-health" OR mHealth OR "m-health" OR telehealth OR "mobile health" OR "digital health" OR "web-based" OR "internet-based" OR "online program*" OR "digital program*" OR "online intervention*" OR "digital intervention*" OR "internet-delivered" OR "mobile app*" OR smartphone* OR "virtual support" OR "remote support")) | 1,740 |
| ProQuest Dissertations & Theses Global | #1 Caregiver | TI,AB(caregiver* OR carer* OR "family caregiver*" OR "informal caregiver*" OR "spouse caregiver*" OR "adult child caregiver*" OR "unpaid caregiver*" OR "primary caregiver*") | 25,622 |
|  | #2 Dementia | TI,AB(Dementia OR "Alzheimer Disease" OR alzheimer* OR "cognitive impairment" OR "major neurocognitive disorder") | 26,118 |
|  | #3 eHealth | TI,AB(Telemedicine OR "Mobile Applications" OR "Remote Consultation" OR eHealth OR "e-health" OR mHealth OR "m-health" OR telehealth OR "mobile health" OR "digital health" OR "web-based" OR "internet-based" OR "online program*" OR "digital program*" OR "online intervention*" OR "digital intervention*" OR "internet-delivered" OR "mobile app*" OR smartphone* OR "virtual support" OR "remote support") | 31,857 |
|  | #4 | TI,AB((caregiver* OR carer* OR "family caregiver*" OR "informal caregiver*" OR "spouse caregiver*" OR "adult child caregiver*" OR "unpaid caregiver*" OR "primary caregiver*") AND (Dementia OR "Alzheimer Disease" OR alzheimer* OR "cognitive impairment" OR "major neurocognitive disorder") AND (Telemedicine OR "Mobile Applications" OR "Remote Consultation" OR eHealth OR "e-health" OR mHealth OR "m-health" OR telehealth OR "mobile health" OR "digital health" OR "web-based" OR "internet-based" OR "online program*" OR "digital program*" OR "online intervention*" OR "digital intervention*" OR "internet-delivered" OR "mobile app*" OR smartphone* OR "virtual support" OR "remote support")) | 52 |
| CINAHL | #1 Caregiver | TI (caregiver* OR carer* OR "family caregiver*" OR "informal caregiver*" OR "spouse caregiver*" OR "adult child caregiver*" OR "unpaid caregiver*" OR "primary caregiver*") OR AB (caregiver* OR carer* OR "family caregiver*" OR "informal caregiver*" OR "spouse caregiver*" OR "adult child caregiver*" OR "unpaid caregiver*" OR "primary caregiver*") | 4,591 |
|  | #2 Dementia | TI (Dementia OR "Alzheimer Disease" OR dementia* OR alzheimer* OR "cognitive impairment" OR "major neurocognitive disorder") OR AB (Dementia OR "Alzheimer Disease" OR dementia* OR alzheimer* OR "cognitive impairment" OR "major neurocognitive disorder") | 4,992 |
|  | #3 eHealth | TI (Telemedicine OR "Mobile Applications" OR "Remote Consultation" OR eHealth OR e-health OR mHealth OR m-health OR telehealth OR telemedicine OR "mobile health" OR "digital health" OR "web-based" OR "internet-based" OR "online program*" OR "digital program*" OR "online intervention*" OR "digital intervention*" OR "internet-delivered" OR "mobile app*" OR smartphone* OR "virtual support" OR "remote support") OR AB (Telemedicine OR "Mobile Applications" OR "Remote Consultation" OR eHealth OR e-health OR mHealth OR m-health OR telehealth OR telemedicine OR "mobile health" OR "digital health" OR "web-based" OR "internet-based" OR "online program*" OR "digital program*" OR "online intervention*" OR "digital intervention*" OR "internet-delivered" OR "mobile app*" OR smartphone* OR "virtual support" OR "remote support") | 4,623 |
|  | #4 | (TI (caregiver* OR carer* OR "family caregiver*" OR "informal caregiver*" OR "spouse caregiver*" OR "adult child caregiver*" OR "unpaid caregiver*" OR "primary caregiver*") OR AB (caregiver* OR carer* OR "family caregiver*" OR "informal caregiver*" OR "spouse caregiver*" OR "adult child caregiver*" OR "unpaid caregiver*" OR "primary caregiver*")) AND (TI (Dementia OR "Alzheimer Disease" OR dementia* OR alzheimer* OR "cognitive impairment" OR "major neurocognitive disorder") OR AB (Dementia OR "Alzheimer Disease" OR dementia* OR alzheimer* OR "cognitive impairment" OR "major neurocognitive disorder")) AND (TI (Telemedicine OR "Mobile Applications" OR "Remote Consultation" OR eHealth OR e-health OR mHealth OR m-health OR telehealth OR telemedicine OR "mobile health" OR "digital health" OR "web-based" OR "internet-based" OR "online program*" OR "digital program*" OR "online intervention*" OR "digital intervention*" OR "internet-delivered" OR "mobile app*" OR smartphone* OR "virtual support" OR "remote support") OR AB (Telemedicine OR "Mobile Applications" OR "Remote Consultation" OR eHealth OR e-health OR mHealth OR m-health OR telehealth OR telemedicine OR "mobile health" OR "digital health" OR "web-based" OR "internet-based" OR "online program*" OR "digital program*" OR "online intervention*" OR "digital intervention*" OR "internet-delivered" OR "mobile app*" OR smartphone* OR "virtual support" OR "remote support")) | 60 |
| PsycINFO | #1 Caregiver | TI(caregiver* OR carer* OR "family caregiver*" OR "informal caregiver*" OR "spouse caregiver*" OR "adult child caregiver*" OR "unpaid caregiver*" OR "primary caregiver*") OR AB (caregiver* OR carer* OR "family caregiver*" OR "informal caregiver*" OR "spouse caregiver*" OR "adult child caregiver*" OR "unpaid caregiver*" OR "primary caregiver*") | 82,968 |
|  | #2 Dementia | TI(dementia OR alzheimer* OR "cognitive impairment" OR "major neurocognitive disorder") OR AB(dementia OR alzheimer* OR "cognitive impairment" OR "major neurocognitive disorder") | 153,626 |
|  | #3 eHealth | TI(eHealth OR e-health OR mHealth OR telehealth OR telemedicine OR "digital health" OR "web-based" OR "internet-based" OR "online intervention*" OR "digital intervention*" OR "mobile app*" OR smartphone*) OR AB(eHealth OR e-health OR mHealth OR telehealth OR telemedicine OR "digital health" OR "web-based" OR "internet-based" OR "online intervention*" OR "digital intervention*" OR "mobile app*" OR smartphone*) | 48,957 |
|  | #4 | (TI(caregiver* OR carer* OR "family caregiver*" OR "informal caregiver*" OR "spouse caregiver*" OR "adult child caregiver*" OR "unpaid caregiver*" OR "primary caregiver*") OR AB (caregiver* OR carer* OR "family caregiver*" OR "informal caregiver*" OR "spouse caregiver*" OR "adult child caregiver*" OR "unpaid caregiver*" OR "primary caregiver*")) AND (TI(dementia OR alzheimer* OR "cognitive impairment" OR "major neurocognitive disorder") OR AB(dementia OR alzheimer* OR "cognitive impairment" OR "major neurocognitive disorder")) AND (TI(eHealth OR e-health OR mHealth OR telehealth OR telemedicine OR "digital health" OR "web-based" OR "internet-based" OR "online intervention*" OR "digital intervention*" OR "mobile app*" OR smartphone*) OR AB(eHealth OR e-health OR mHealth OR telehealth OR telemedicine OR "digital health" OR "web-based" OR "internet-based" OR "online intervention*" OR "digital intervention*" OR "mobile app*" OR smartphone*)) | 314 |
| ClinicalTrials.gov | Search | Condition or disease: dementia OR Alzheimer  Other terms: caregiver OR "family caregiver" OR informal caregiver OR carer  Intervention: telehealth OR telemedicine OR ehealth OR mhealth OR "digital health" OR "web-based" OR "online intervention" OR "mobile app" | 194 |

Note: All searches were performed on March 10, 2026.

Table S2. Characteristics of included randomized controlled trials evaluating eHealth interventions for informal caregivers of people with dementia (detailed edition; k = 35).

| Study | Sample Size (Analyzed/Total) | Attrition Rate | Caregiver Summary | Care Recipient | Delivery/Duration | Intervention Component | Theory | Comparator | Adherence/Engagement | Outcome |
| --- | --- | --- | --- | --- | --- | --- | --- | --- | --- | --- |
| Beauchamp et al [[27](https://jmir.kriyadocs.com/web_preview?doi=78568&project=jmir&customer=jmir#ref27)], 2005 (USA) | 299/307 (I: 150, C: 149) | 2.6% | 46.9 ± 12.2 (73.0% F) – Adult Child 67% | Mixed (Clinical diagnosis, all stages) | Web-based/Asynchronous/4 weeks | Self-Guided: Multi-component (Education & CBT-based skills) | SCM | Inert | Passive Tracking/Variable: Mean 32.2 min; 41% >1 visit | *Caregiver Strain (CSI) Depressive Symptoms (CES-D) |
| Gitlin et al [[28](https://jmir.kriyadocs.com/web_preview?doi=78568&project=jmir&customer=jmir#ref28)], 2010 (USA) | 239/272 (I: 117, C: 122) | 12.1% | 66.3 ± 12.2 (82.0%F) - Spouse 51% | Mixed (Clinical diagnosis, all stages) | Video/Tele (VC)/Synchronous/24 weeks | Human-Supported: ACT^a^: Identifying/modifying triggers, problem-solving training (DICE-like), stress reduction, medical screening, and provision of assistive devices. | SHPM | Inert | Active Monitoring/High: Mean 8.6 occupational therapy sessions, 2.0 Nurse contacts, and 2.7 maintenance calls completed. | Caregiver Burden (ZBI) Depressive Symptoms (CES-D) |
| Kwok et al [[29](https://jmir.kriyadocs.com/web_preview?doi=78568&project=jmir&customer=jmir#ref29)], 2013 (China) | 38/42 (I: 18, C: 20) | 9.5% | IG: 51–60 (Median); CG: 41–50 (Median) (71.1% F) – Adult Child 81.6% | Mixed (Clinical diagnosis, all stages) | App/IM (Mobile)/Synchronous/12 weeks | Self-Guided: Psychoeducation | PEF | Information | Active Monitoring/High: All participants completed | Caregiver Burden (ZBI) |
| Torkamani et al [[30](https://jmir.kriyadocs.com/web_preview?doi=78568&project=jmir&customer=jmir#ref30)], 2014 (UK, Spain, Greece) | 57/60 (I: 27, C: 30) | 5.0% | IG: 57.6 ± 12.5; CG: 63.9 ± 14.7 (45.0% F) – Mixed | Mild to Moderate (MMSE: 9–21) | Web-based/Asynchronous/24 weeks | Self-Guided: Monitoring/Alerts (MY TASKS) | None explicit | Active | Active Monitoring/High: Self-scheduled; 64% more confident | Caregiver Burden (ZBI) |
| Kales et al [[31](https://jmir.kriyadocs.com/web_preview?doi=78568&project=jmir&customer=jmir#ref31)], 2018 (USA) | 56/57 (I: 26, C: 30) | 1.8% | 65.9 ± 14.0 (75.0% F) – Spouse 49% | Moderate (MMSE: 16.5 ± 8.3) | App/IM (Mobile)/Asynchronous/4 weeks | Human-Supported: Guided Symptom Management (WCA tool) | DICE™ | Inert | Active Monitoring/High: Weekly calls; engagement tracked | *Depressive Symptoms (CES-D) Caregiver Burden (ZBI) |
| Meichsner et al [[32](https://jmir.kriyadocs.com/web_preview?doi=78568&project=jmir&customer=jmir#ref32)], 2019 (Germany) | 30/37 (I: 15, C: 15) | 16.22% | 62.11 ± 9.67 (78.4%F) - Spouse 73% | Moderate to Severe (GDS stages 5–6) | Web-based/Asynchronous/8 weeks | Human-Supported: Tele.TAnDem.online: 10 CBT modules including problem analysis, psychoeducation, restructuring cognitions, and self-care. | CBT + ACT + Resource Activation | Inert | Active Monitoring/High: High: Mean 12.4 messages sent; Mean 62.19 mins spent writing per message. | *Caregiver Burden (VAS) *Depressive Symptoms (CES-D) |
| Metcalfe et al [[33](https://jmir.kriyadocs.com/web_preview?doi=78568&project=jmir&customer=jmir#ref33)], 2019 (England, France, Germany) | 58/61 (I: 29, C: 29) | 4.90% | IG: 57.6 ± 10.5; CG: 57.2 ± 9.9 (60.7%F) - Spouse General | Young Onset Dementia (AD or FTD) | Web-based/Asynchronous/6 weeks | Self-Guided: RHAPSODY: 7 modules (Medical explanations, symptom management, relationship changes, care/support, and self-care) | REACH II; TAM | Inert | Passive Tracking/Variable: Average 7.5 visits over 6 weeks; 31% of content consulted | Caregiver Burden (BSFC-10) Depressive Symptoms (PSS-10) |
| Williams et al [[34](https://jmir.kriyadocs.com/web_preview?doi=78568&project=jmir&customer=jmir#ref34)], 2019 (USA) | 84/107 (I: 43, C: 41) | 21.50% | 64.2 ± 12.8 (71.2%F) - Spouse 66.3% | Moderate (53.9%) | Video/Tele (VC)/Hybrid/12 weeks | Human-Supported: FamTechCare: Video-recording of challenging care (buffering technology), expert interdisciplinary review, and weekly tailored telephone feedback | NDCBM | Active | Active Monitoring/High: Mean: 21.0 ± 27.9 videos submitted (Range: 1–172); Mean: 9.1 ± 2.5 phone calls | *Caregiver Burden (ZBI) *Depressive Symptoms (CES-D) |
| James et al [[35](https://jmir.kriyadocs.com/web_preview?doi=78568&project=jmir&customer=jmir#ref35)], 2021 (USA) | 10/28 (I: 5, C: 5) | 64.3% | 60.3 ± 7.4 (73.7% F) – Spouse 63.2% | Moderate (Katz ADL: 3.30) | App/IM (Mobile)/Asynchronous/2 weeks | Self-Guided: Mind–Body Exercises & Heart Rate Variability Monitoring (HeartMath/Welltory) | None explicit | Inert | Active Monitoring/High: all completers submitted logs | Caregiver Burden (ZBI) |
| Baruah et al [[36](https://jmir.kriyadocs.com/web_preview?doi=78568&project=jmir&customer=jmir#ref36)], 2021 (India) | 55/151 (I: 29, C: 26) | 63.6% | IG: 53.03 ± 15.31; CG: 45.88 ± 11.56 (46.0%F) - Adult Child 60% | Mixed (Clinical diagnosis, all stages) | Web-based/Asynchronous/12 weeks | Self-Guided: iSupport-India: 23 lessons covering dementia info, self-care, everyday care, and behavior management | WHO iSupport | Information | Passive Tracking/Variable: Low: 70.3% visited once; 40.5% did 1 lesson; only 16.2% completed recommended 5 lessons | *Caregiver Burden (ZBI) *Depressive Symptoms (CES-D) |
| Fossey et al [[37](https://jmir.kriyadocs.com/web_preview?doi=78568&project=jmir&customer=jmir#ref37)], 2021 (UK) | 208/638 (CCBT = 53, CCBT+Phone = 101, C = 54) | 67.4% | 59.9 ± 12.2 (85.0%F) - Mixed | Mixed (Clinical diagnosis, all stages) | Web-based/Asynchronous/26 weeks | Human-Supported: CCBT: 20 sessions on thoughts, feelings, behavior, and goal setting. Education: 20 sessions on dementia facts, care skills, and well-being. | CBT + Psychoeducation | Active | Active Monitoring/Variable: 26% completed ≥80% of sessions; 46% completed ≥1 session. Telephone arm had higher engagement. | Depressive Symptoms Caregiver Burden (RSS) |
| Teles et al [[38](https://jmir.kriyadocs.com/web_preview?doi=78568&project=jmir&customer=jmir#ref38)], 2022 (Portugal) | 31/42 (I: 11, C: 20) | 26.2% | 53.6 ± 13.0 (78.6% F) – Adult Child 73.8% | Mixed (Clinical diagnosis, all stages) | Web-based/Asynchronous/12 weeks | Self-Guided: Online Knowledge/Skills Training (iSupport-Portugal) | WHO iSupport | Information | Passive Tracking/Variable: Median 13 lessons; 39% active at 3 months | *Caregiver Burden (ZBI) Depressive Symptoms (CES-D) |
| Hepburn et al [[39](https://jmir.kriyadocs.com/web_preview?doi=78568&project=jmir&customer=jmir#ref39)], 2022 (USA) | 261/343 (I: 96, C: 165) | 23.8% | 64.6 ± 11.2 (70.5% F) – Spouse 65.9% | Mixed (Clinical diagnosis, all stages) | Video/Tele (VC)/Synchronous/7 weeks | Human-Supported: Psychoeducation & Group Coaching | SCT; SCM | Active | Active Monitoring/Undefined: Monitored by “Navigators”; attrition as expected | *Caregiver Burden (ZBI) Depressive Symptoms (HADS-D) |
| Bodenstein [[40](https://jmir.kriyadocs.com/web_preview?doi=78568&project=jmir&customer=jmir#ref40)], 2022 (Canada) | 30/35 (I: 16, C: 14) | 16.7% | 73.0 ± 12.9 (66.7%F) - Mixed | Mixed (Clinical diagnosis, all stages) | Video/Tele (VC)/Synchronous/8 weeks | Human-Supported: Virtual Mindful Chair Yoga (MCY): Meditation, gentle yoga postures (asanas), breathing (pranayama), and mindfulness exercises | MBI | Inert | Active Monitoring/High: 83.3% completion; encouraged 15 min/day home practice | Caregiver Burden (ZBI) Depressive Symptoms (PHQ-9) |
| Han et al [[41](https://jmir.kriyadocs.com/web_preview?doi=78568&project=jmir&customer=jmir#ref41)], 2023 (USA) | 19/19 (I: 9, C: 10) | 5.30% | 18–78 (100.0%F) - Adult Child 78.9% | Moderate (58%) | Video/Tele (VC)/Synchronous/8 weeks | Human-Supported: Videoconferencing ACT^b^ (6 processes) + Behavioral Activation (activity scheduling) + Psychoeducation materials | ACT + BA | Information | Active Monitoring/High: 100% session attendance in the ACT group | Caregiver Burden (ZBI) Depressive Symptoms (DASS-21) |
| Rhodus et al [[42](https://jmir.kriyadocs.com/web_preview?doi=78568&project=jmir&customer=jmir#ref42)], 2023 (USA) | 25/30 (HARMONY = 9, Standard = 9, C: 7) | 16.70% | 69.0 ± 11.7 (63.3%F) - Mixed | Mixed (Clinical diagnosis, all stages) | Video/Tele (VC)/Synchronous/6 weeks | Human-Supported: HARMONY: Guided discovery, sensory-based environmental cueing, individualized goal selection, and sensory tool kits | PEOP | Active | Active Monitoring/Variable: M visits: HARMONY = 5.4; Standard = 4.9; Control = 4.6 | Caregiver Burden (ZBI) |
| Rodriguez et al [[43](https://jmir.kriyadocs.com/web_preview?doi=78568&project=jmir&customer=jmir#ref43)], 2023 (USA) | 45/53 (I: 23, C: 22) | 15% | 62.9 ± 13.2 (77.4%F) - Mixed | Mixed (Clinical diagnosis, all stages) | App/IM (Mobile)/Asynchronous/24 weeks | Human-Supported: Brain CareNotes: 24/7 psychoeducation, H-ABC Monitor assessments, automated "CareNotes" advice cards, care coach messaging | CCM | Active | Active Monitoring/High: Usage rates (daily/weekly/monthly) collected; Intent-to-use measured | Caregiver Burden (NPI-Caregiver Distress) |
| Hu et al [[44](https://jmir.kriyadocs.com/web_preview?doi=78568&project=jmir&customer=jmir#ref44)], 2024 (China) | 60/60 (I: 30, C: 30) | 0.0% | IG: 54.8 ± 8.27; CG: 50.9 ± 8.91 (56.7% F) – Mixed | Mild to Moderate (MMSE: >17) | App/IM (Mobile)/Synchronous/8 weeks | Human-Supported: Mindfulness-Based Stress Reduction (MBSR) | MBSR | Information | Active Monitoring/Undefined: WeChat + peer encouragement | *Caregiver Burden (ZBI) Depressive Symptoms (DASS-21) |
| Salehinejad et al [[45](https://jmir.kriyadocs.com/web_preview?doi=78568&project=jmir&customer=jmir#ref45)], 2024 (Iran) | 47/50 (I: 24, C: 23) | 6.0% | 41–50 (Majority) (62.0% F) – Adult Child 68% | Moderate to Severe (Qualitative) | Web-based/Asynchronous/8 weeks | Self-Guided: Education + Behavioral Management | None explicit | Active | Active Monitoring/High: Website logins tracked; sessions validated | Caregiver Burden (ZBI) |
| Xie et al [[46](https://jmir.kriyadocs.com/web_preview?doi=78568&project=jmir&customer=jmir#ref46)], 2024 (China) | 66/72 (I: 33, C: 33) | 8.3% | 57.74 ± 3.93 (86.0% F) – Mixed | Mixed (Clinical diagnosis, all stages) | Web-based/Asynchronous/24 weeks | Self-Guided: Multidisciplinary Caregiver Training (Nurse-led) | NL-MTSP | Information | Active Monitoring/High: >80% completion; screen time tracked | Caregiver Burden (ZBI) |
| Jain [[47](https://jmir.kriyadocs.com/web_preview?doi=78568&project=jmir&customer=jmir#ref47)], 2024 (India) | 16/30 (I: 8, C: 8) | 46.70% | IG: 29.0 (Median); CG: 31.5 (Median) (31.3%F) - Mixed | Mild to Moderate (DSRS < 37) | Video/Tele (VC)/Synchronous/3 weeks | Human-Supported: Brief CBT (Psychoeducation, Behavioral strategies, Cognitive reframing) + Mindfulness (Raisin, Breathing, Body scan, Walking, 3-min space). | START; MBCT | Inert | Active Monitoring/High: Session-specific homework review; Behavior/Thought records | *Caregiver Burden (ZBI) |
| Song et al [[48](https://jmir.kriyadocs.com/web_preview?doi=78568&project=jmir&customer=jmir#ref48)], 2024 (USA) | 27/30 (I: 13, C: 14) | 10.00% | 67.0 ± 10.9 (93.3%F) - Spouse 60% | Mixed (Clinical diagnosis, all stages) | Video/Tele (VC)/Synchronous/5 weeks | Human-Supported: Care2Sleep: CBT-I (sleep hygiene, compression, stimulus control, relaxation), daily walking, light exposure, and "A-B-C" problem-solving for dementia behaviors | CBT-I | Information | Active Monitoring/High: Adherence measured via CP diary and session notes; Sleep schedule adherence: 69% (CP) | Caregiver Burden (ZBI) |
| Yuan et al [[49](https://jmir.kriyadocs.com/web_preview?doi=78568&project=jmir&customer=jmir#ref49)], 2025 (China) | 106/120 (I: 51, C: 55) | 11.7% | IG: 57 ± 12.68; CG: 52 ± 13.66 (60.8% F) – Adult Child 70% | Mixed (Clinical diagnosis, all stages) | App/IM (Mobile)/Synchronous/20 weeks | Human-Supported: iSupport Modules + Follow-Up Support | SST; CFIR | Inert | Passive Tracking/Undefined: Platform learning data: I 23.15 vs C 3.90 | Caregiver Burden (ZBI) |
| Nguyen et al [[50](https://jmir.kriyadocs.com/web_preview?doi=78568&project=jmir&customer=jmir#ref50)], 2025 (Vietnam) | 57/60 (I: 27, C: 30) | 5.0% | IG: 56.1 ± 10.1; CG: 54.6 ± 8.1 (78.3% F) – Adult Child 65% | Severe (80.0%–86.7% severe) | App/IM (Mobile)/Asynchronous/7 weeks | Human-Supported: Psychoeducation + Facilitator-Supported Discussion | None explicit | Information | Active Monitoring/High: >85% weekly engagement; all completed ratings | Caregiver Burden (ZBI) Depressive Symptoms (DASS-21) |
| Nguyen et al [[51](https://jmir.kriyadocs.com/web_preview?doi=78568&project=jmir&customer=jmir#ref51)], 2025 (Vietnam) | 161/172 (I: 80, C: 81) | 6.4% | IG: 52.5 ± 9.2; CG: 51.5 ± 9.4 (72.1% F) – Adult Child 77.9% | Severe (Approx. 82% severe) | App/IM (Mobile)/Asynchronous/7 weeks | Human-Supported: Video-Based Psychoeducation & Messaging Support | WHO iSupport | Information | Passive Tracking/High: Low attrition; no adverse effects | *Depressive Symptoms (DASS-21) Caregiver Burden (ZBI) |
| Han et al [[52](https://jmir.kriyadocs.com/web_preview?doi=78568&project=jmir&customer=jmir#ref52)], 2025 (USA) | 33/33 (I: 16, C: 17) | 15.2% | 55.2 ± 12.7 (87.9% F) – Non-spousal 76% | Mixed (Mild 15%, Mod 49%, Sev 27%) | Video/Tele (VC)/Synchronous/10 weeks | Self-Guided: ACT^b^-Based Intervention | ACT | Information | Active Monitoring/High: Full attendance; coach logs maintained | *Depressive Symptoms (PHQ-9) Caregiver Burden (ZBI) |
| Nichols et al [[53](https://jmir.kriyadocs.com/web_preview?doi=78568&project=jmir&customer=jmir#ref53)], 2025 (USA) | 110/110 (I: 56, C: 54) | 0.0% | 65.1 ± 10.7 (92.7% F) – Spouse 77.5% | Severe (High functional dependency: >90% IADL) | App/IM (Mobile)/Synchronous/12 weeks | Human-Supported: Problem-Solving & Coping Skills | REACH II | Inert | Active Monitoring/High: Personalized coaching; in-app modules | *Caregiver Burden (ZBI) Depressive Symptoms (PHQ-9) |
| Stevens et al [[54](https://jmir.kriyadocs.com/web_preview?doi=78568&project=jmir&customer=jmir#ref54)], 2025 (USA) | 167/240 (I: 79, C: 88) | 30.4% | 64.76 ± 12.06 (80.0% F) – Adult Child 52% | Mixed (AD8: 7.54 ± 0.97) | Web-based/Asynchronous/24 weeks | Human-Supported: Online Psychoeducation + Skills + Caregiver Network (GP4C) | REACH II | Active | Passive Tracking/Variable: GP4C 14 logins/5.5 h; R4C 5 logins/1.9 h | *Caregiver Burden (ZBI) Depressive Symptoms (CES-D) |
| Pfaff et al [[55](https://jmir.kriyadocs.com/web_preview?doi=78568&project=jmir&customer=jmir#ref55)], 2025 (Germany) | 170/192 (I: 83, C: 87) | 11.50% | IG: 67.3 ± 11.6; CG: 63.6 ± 12.3 (75.0%F) - Spouse 58.3% | Mixed (Clinical diagnosis, all stages) | Video/Tele (VC)/Hybrid/24 weeks | Human-Supported: GAIN: IT-based self-assessment, nurse-led needs assessment, individualized care plans, algorithm-based expert system | RBES; DelpHi-MV | Inert | Active Monitoring/High: 88 participants received the allocated intervention; monthly follow-up calls used to monitor implementation | Caregiver Burden (ZBI) |
| Windle et al [[56](https://jmir.kriyadocs.com/web_preview?doi=78568&project=jmir&customer=jmir#ref56)], 2025 (UK) | 352/352 (I = 175, C = 177) | 25.30% | 62.2 ± 11.6 (79.6%F) - Adult Child 50.3% | Mixed (Clinical diagnosis, all stages) | Web-based/Asynchronous/24 weeks | Self-Guided: iSupport: 5 modules (Introduction, Being a Carer, Caring for Me, Everyday Care, Behaviour Changes) | CBT + Psychoeducation | Information | Passive Tracking/Variable: Median 4 logons (IQR 1-10); Median 49 mins total use (IQR 5-104) | *Caregiver Burden (ZBI) *Depressive Symptoms (CES-D) |
| Sun et al [[57](https://jmir.kriyadocs.com/web_preview?doi=78568&project=jmir&customer=jmir#ref57)], 2026 (China) | 201/250 (I: 98, C: 103) | 19.6% | 42.91 ± 13.83 (51.2% F) – Adult Child 63.2% | Mixed (Clinical diagnosis, all stages) | App/IM (Mobile)/Asynchronous/12 weeks | Self-Guided: Personalized Care Plans & Expert Consultation | None explicit | Inert | Passive Tracking/Undefined: Weekly assessment advised; auto-tracking | *Caregiver Burden (ZBI) Depressive Symptoms (SDS) |
| Yuan et al [[58](https://jmir.kriyadocs.com/web_preview?doi=78568&project=jmir&customer=jmir#ref58)], 2026 (Singapore) | 52/53 (I: 27, C: 26) | 1.9% | 56.5 ± 8.3 (83.0% F) – Adult Child 79.2% | Mixed (Clinical diagnosis, all stages) | App/IM (Mobile)/Asynchronous/4 weeks | Self-Guided: Knowledge Base & Peer Support Forum | MTM of Stress and Coping | Inert | Passive Tracking/High: Avg 83.2 min; 54% weekly; 85% ≥1 journal entry | *Depressive Symptoms (CES-D) Caregiver Burden (ZBI) |
| Brijnath et al [[59](https://jmir.kriyadocs.com/web_preview?doi=78568&project=jmir&customer=jmir#ref59)], 2026 (Australia) | 93/116 (I: 47, C: 46) | 19.8% | 54.80 ± 13.33 (77.4%F) - Adult Child 67.8% | Mixed (Clinical diagnosis, all stages) | Web-based/Asynchronous/12 weeks | Self-Guided: 'Draw-Care' (Adapted WHO iSupport Lite): 6 animated films and 10 tipsheets covering seeking support, self-care, care continuity, responding to change, flexibility, and communication | WHO iSupport | Inert | Passive Tracking/High: Median session time 10.82 min (Total trial); median 18.83 min (First 3 months); High engagement relative to other iSupport trials | *Caregiver Burden (ZBI) Depressive Symptoms (CES-D) |
| Cheng and Ng [[60](https://jmir.kriyadocs.com/web_preview?doi=78568&project=jmir&customer=jmir#ref60)], 2026 (43 countries) | 274/441 (I: 132, C: 142) | 31.4% | 54.58 ± 10.83 (85.4%F) - Adult Child 65.7% | Mixed (Clinical diagnosis, all stages) | Web-based/Synchronous/12 weeks | Self-Guided: PDC30: Guidebook, AI-chatbot (GPT-4o), relaxation/CBT apps, bonding apps, graphic novel | BFT | Inert | Passive Tracking/Variable: Used website several times weekly; reminders sent after 3 days inactivity | *Depressive Symptoms (PHQ-9) Caregiver Burden (ZBI) |
| Durepos et al [[61](https://jmir.kriyadocs.com/web_preview?doi=78568&project=jmir&customer=jmir#ref61)], 2026 (Canada) | 29/30 (I: 14, C: 15) | 3.3% | 55.43 ± 9.86 (86.7%F) - Adult Child 56.7% | Mixed (Clinical diagnosis, all stages) | Video/Tele (VC)/Synchronous/16–24 weeks | Human-Supported: Acceptance and Commitment Therapy (ACT^b^): Fostering acceptance, mindfulness, clarifying values, and behavior change. | ACT | Information | Active Monitoring/Variable: 62.5% completed 6–8 sessions within the study timeline. | Caregiver Burden (ZBI) Depressive Symptoms (DASS-21) |

*Denotes the primary outcome as defined in the original study. Abbreviations: ACT^a^: Advancing Caregiver Training; ACT^b^: Acceptance and Commitment Therapy; AD: Alzheimer's Disease; AD8: Ascertain Dementia 8; ADL: Activities of Daily Living; ACT: Acceptance and Commitment Therapy; BA: Behavioral Activation; BFT: Behavioral Family Therapy; BIQ: Behavioral Intention Questionnaire; BSFC-10: Burden Scale for Family Caregivers; CBT: Cognitive Behavioral Therapy; CBT-I: Cognitive Behavioral Therapy for Insomnia; CCM: Collaborative Care Model; CES-D: Center for Epidemiologic Studies Depression Scale; CFIR: Consolidated Framework for Implementation Research; CG: Control Group; CSI: Caregiver Strain Index; DASS-21: Depression, Anxiety and Stress Scale; DelpHi-MV: Dementia Care Management in Northwestern Germany (study acronym); DICE™: Describe–Investigate–Create–Evaluate; DSRS: Dementia Severity Rating Scale; F: Female; FTD: Frontotemporal Dementia; GDS: Global Deterioration Scale; GP4C: GamePlan4Care; HADS: Hospital Anxiety and Depression Scale; IADL: Instrumental Activities of Daily Living; IG: Intervention Group; IM: Instant Messaging; MBI: Mindfulness-Based Intervention; MBCT: Mindfulness-Based Cognitive Therapy; MBSR: Mindfulness-Based Stress Reduction; MMSE: Mini-Mental State Examination; MTM: Modified Transactional Model; NDCBM: Need-based Dementia Compromised Behavior Model; NL-MTSP: nurse-led multidisciplinary team program; NPI-Q: Neuropsychiatric Inventory Questionnaire (Caregiver Distress subscale); PEF: Psychoeducational Framework; PEOP: Person–Environment–Occupation–Performance; PHQ-9: Patient Health Questionnaire; PSS-10: Perceived Stress Scale; R4C: Resources4Care; RBES: Rule-Based Expert System; RCT: Randomized Controlled Trial; REACH II: Resources for Enhancing Alzheimer’s Caregiver Health II; RSS: Relative Stress Scale; SCM: Stress and Coping Model; SCT: Social Cognitive Theory; SDS: Zung Self-Rating Depression Scale; SHPM: Stress Health Process Model; SST: Social Support Theory; TAM: Technology Acceptance Model; VAS: Visual Analog Scale; VC: Videoconferencing; WHO iSupport: World Health Organization iSupport Program; ZBI: Zarit Burden Interview.

Table S3. Comparison of primary meta-analysis and sensitivity analysis.

| **Analysis** | **N _Studies_** | **N _Participants_** | **SMD (95% CI)** | ***P*-value** | I^2^ **(%)** |
| --- | --- | --- | --- | --- | --- |
| Caregiver Burden | 35 | 3,388 | -0.26 [−0.42, −0.10] | .002 | 73.6% |
| Sensitivity | 27 | 2,885 | -0.31 [-0.50, -0.13] | .002 | 77.0% |
| Depressive Symptoms | 23 | 2,467 | -0.27 [−0.53, −0.01] | .042 | 85.7% |
| Sensitivity | 19 | 2,059 | -0.31 [-0.63, 0.01] | .054 | 88.1% |

| **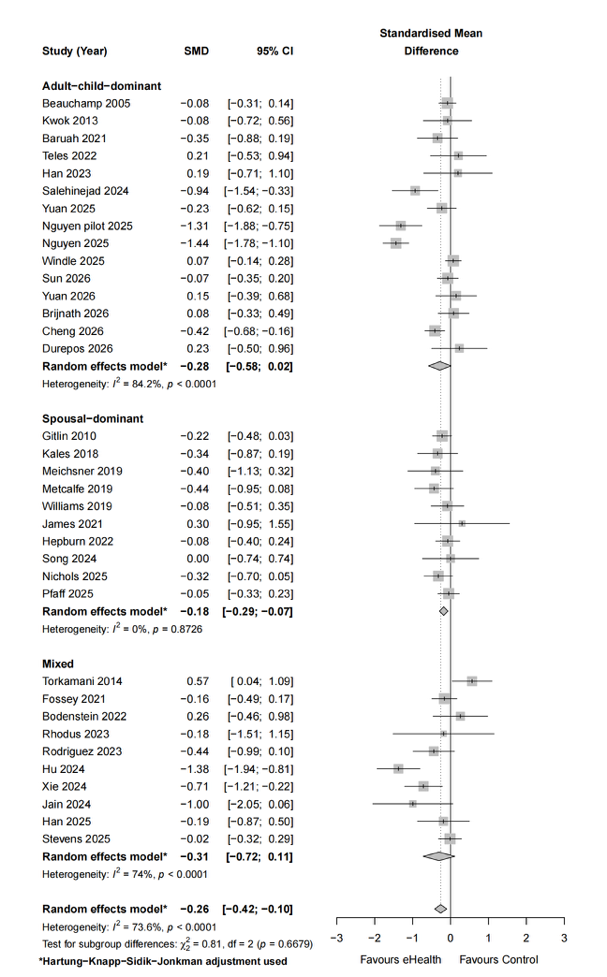** | **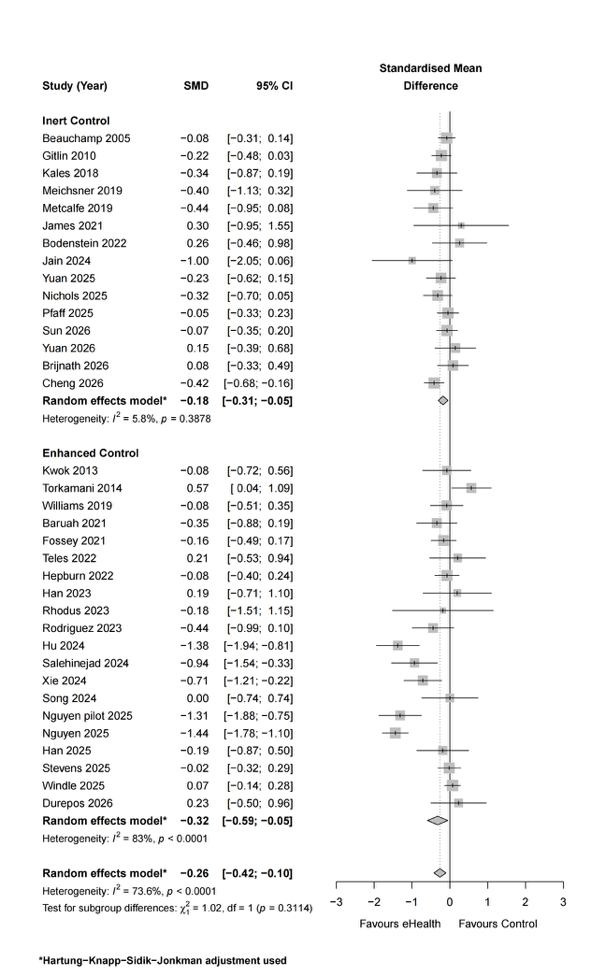** |
| --- | --- |
| Subgroup (a): Caregiver Dominant Type | Subgroup (b): Comparator |
| **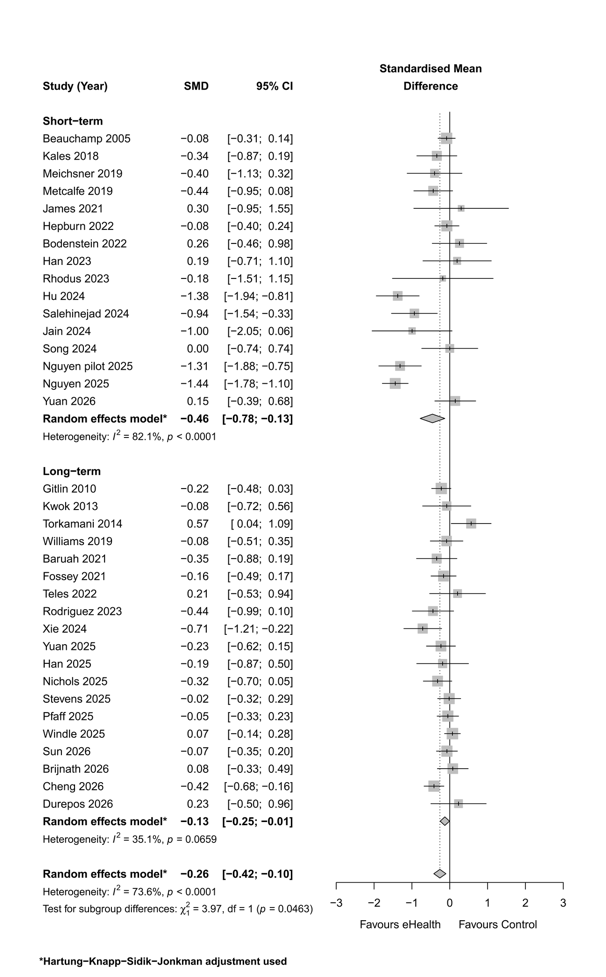** | **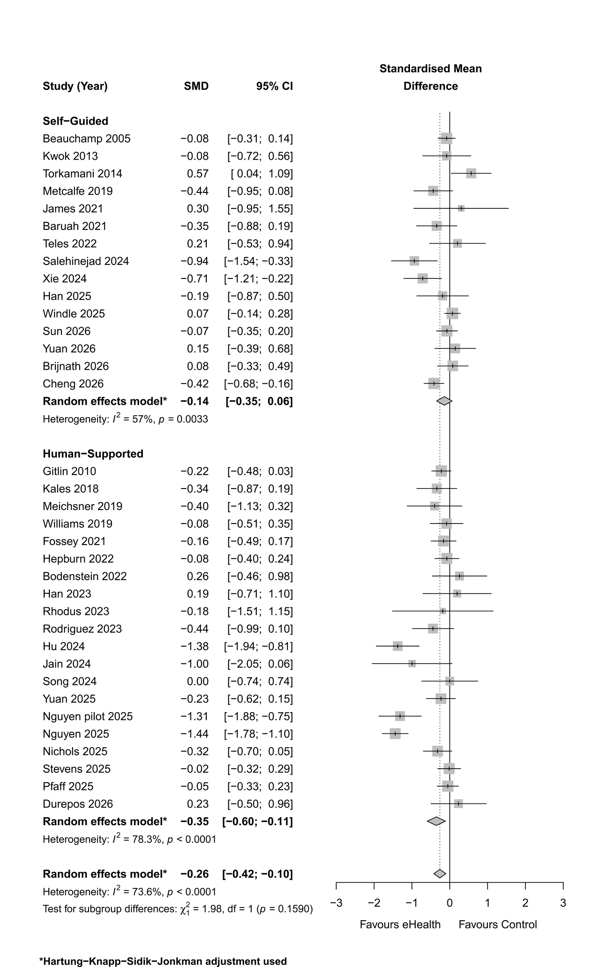** |
| Subgroup (c): Intervention Duration | Subgroup (d): Guidance Category |
| 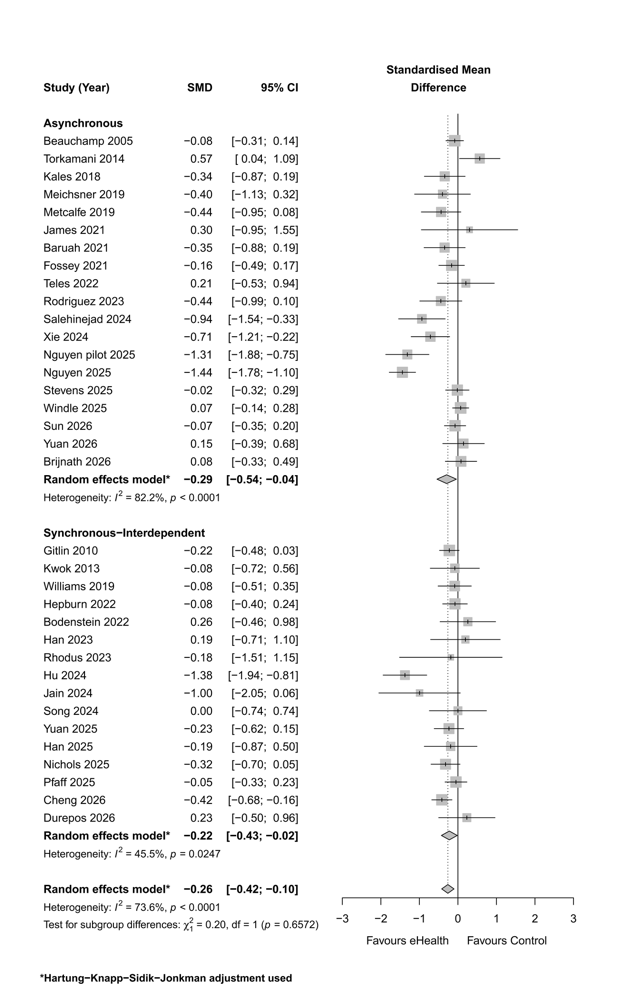 | **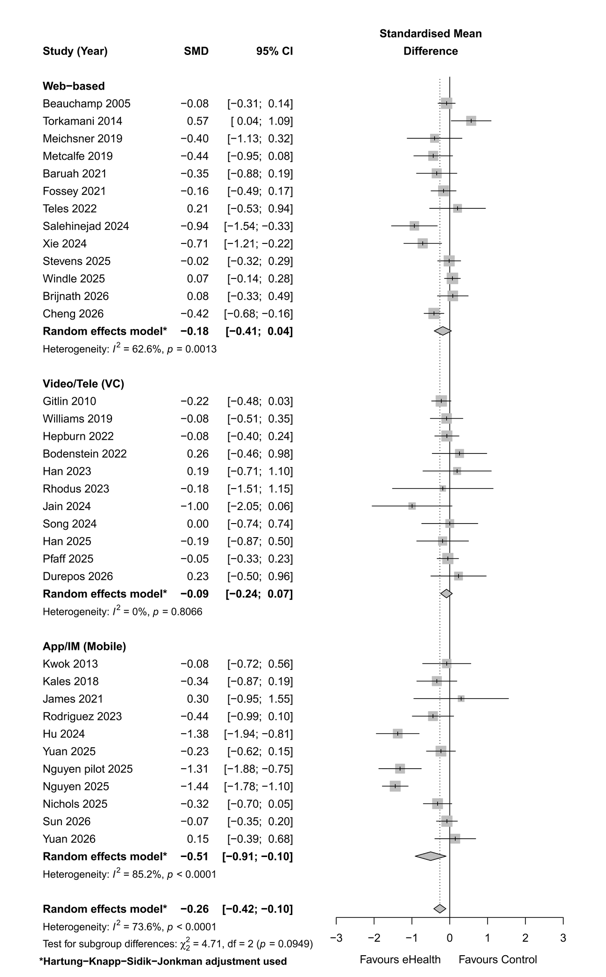** |
| Subgroup (e): Intervention Interaction | Subgroup (f): Modality |
| **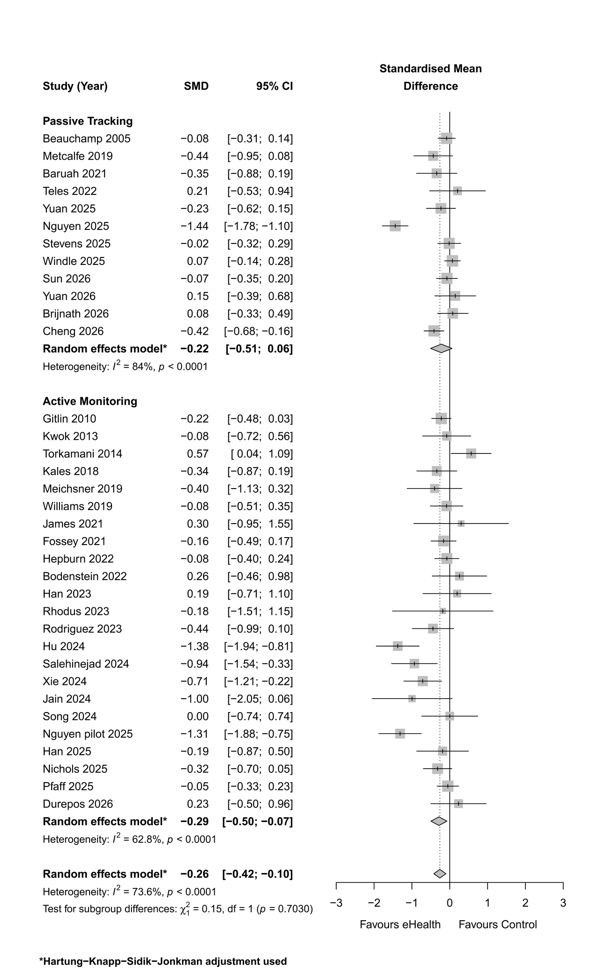** | **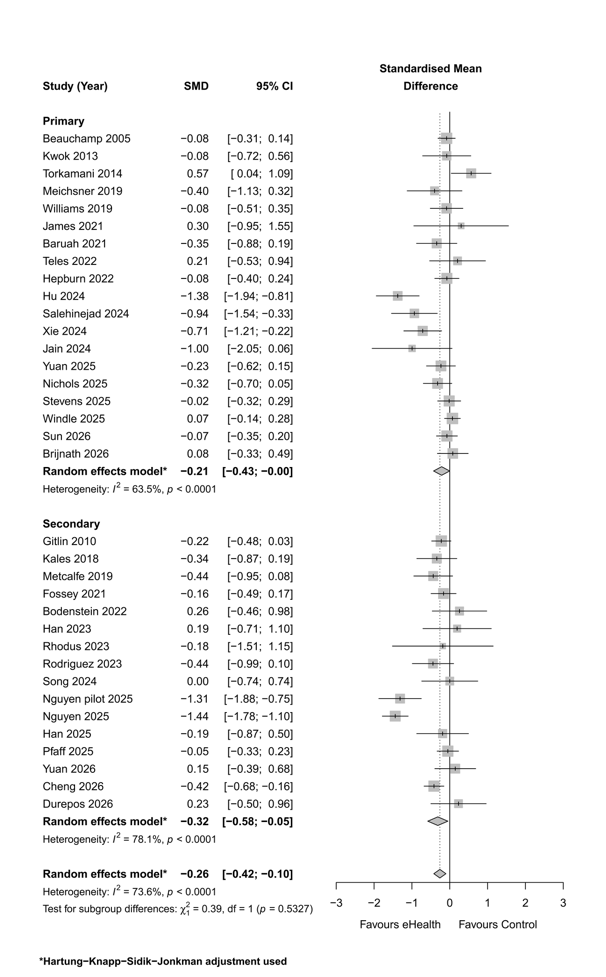** |
| Subgroup (g): Monitoring | Subgroup (h): Outcome Hierarchy |

Figure S1. Forest plots of pre-specified subgroup analyses examining the impact of eHealth interventions on dementia caregiver burden across various moderators.

| **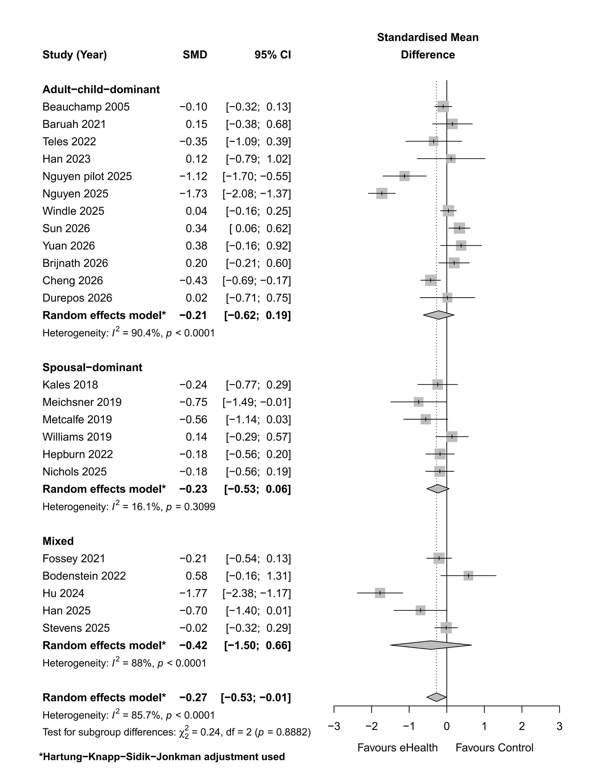** | **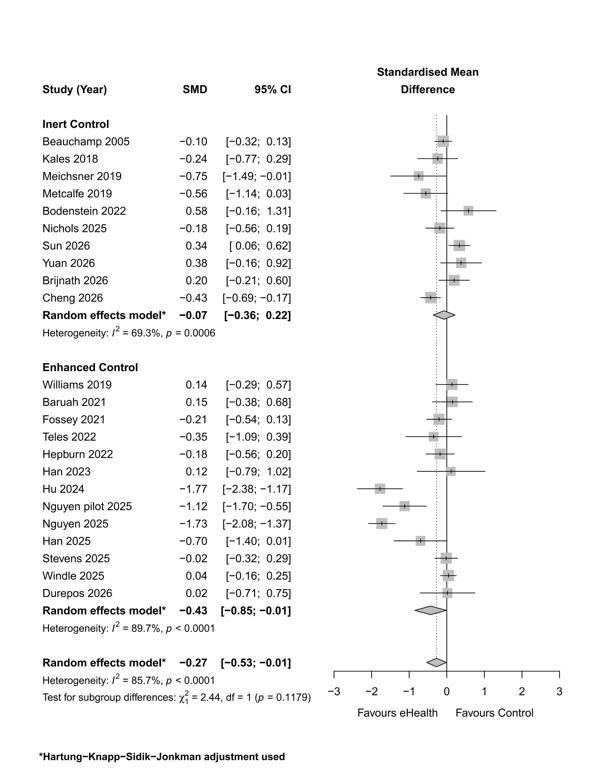** |
| --- | --- |
| Subgroup (a): Caregiver Dominant Type | Subgroup (b): Comparator |
| **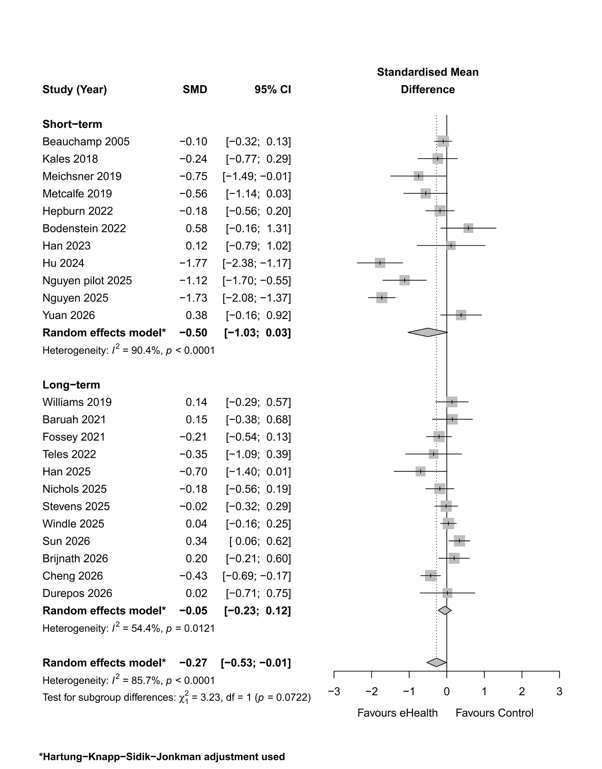** | **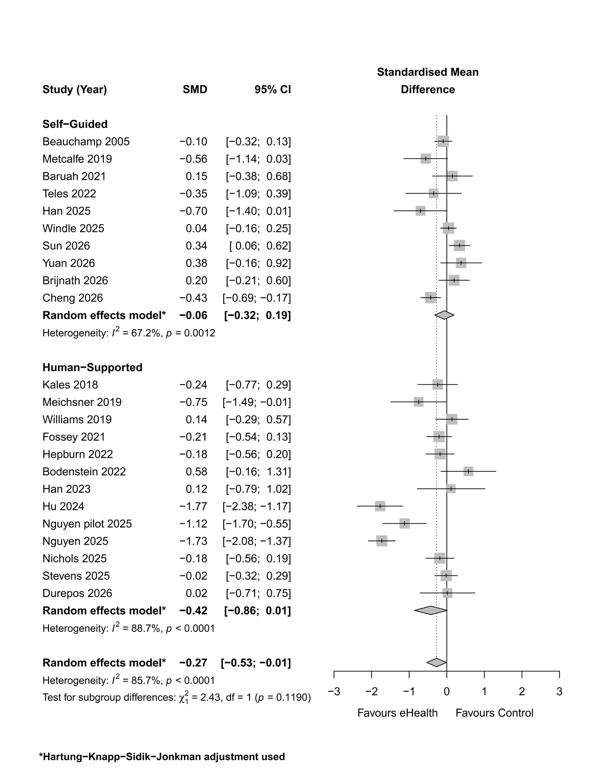** |
| Subgroup (c): Intervention Duration | Subgroup (d): Guidance Category |
| 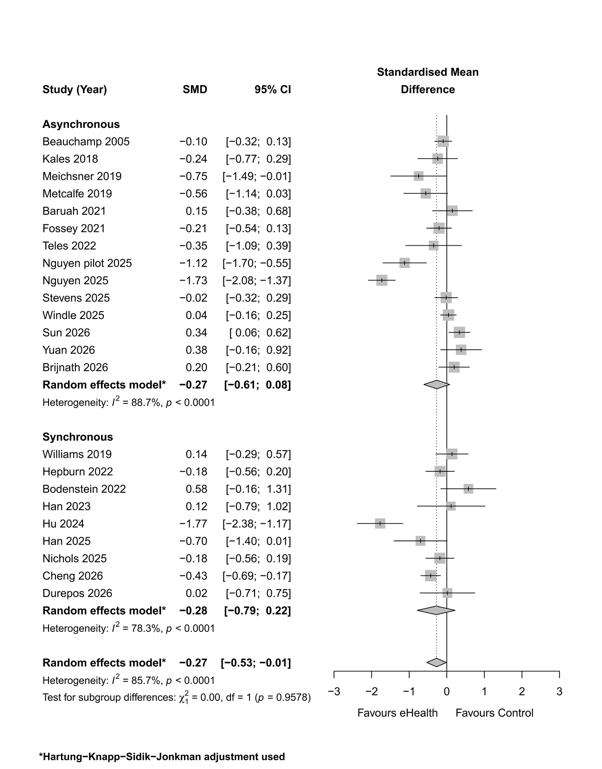 | **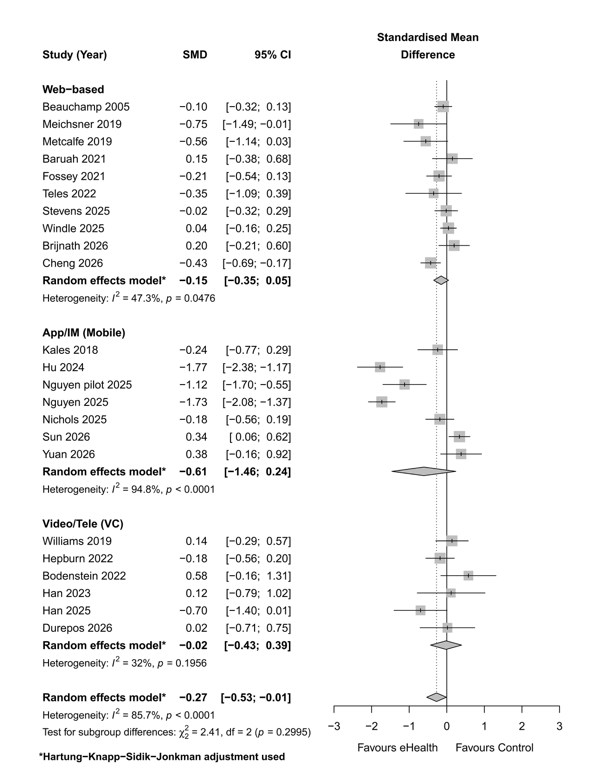** |
| Subgroup (e): Intervention Interaction | Subgroup (f): Modality |
| **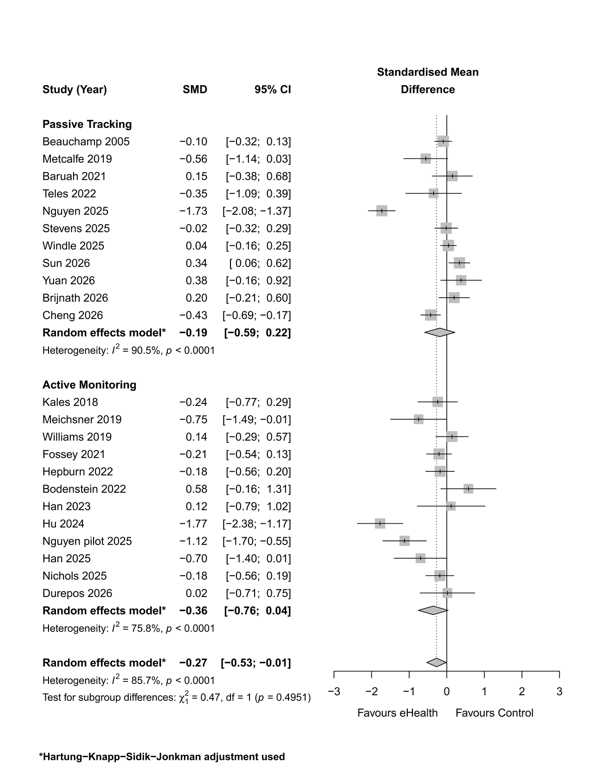** | **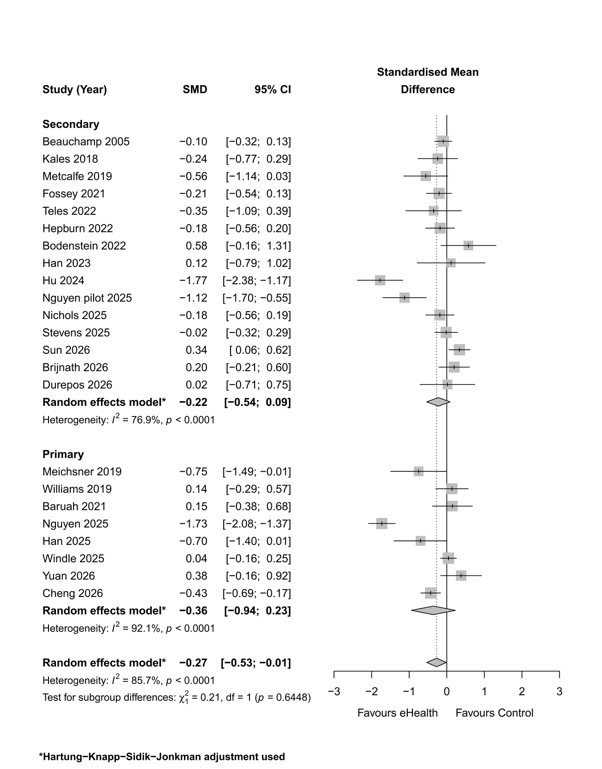** |
| Subgroup (g): Monitoring | Subgroup (h): Outcome Hierarchy |

Figure S2. Subgroup analyses for depressive symptoms across intervention, caregiver, and outcome characteristics.
