# Supplementary material for: Effectiveness of eHealth Interventions in Alleviating Burden on Informal Caregivers of People With Dementia: Systematic Review and Meta-Analysis of Randomized Controlled Trials
Source: J Med Internet Res. 2026 Jun 3;28:e78568. doi: 10.2196/78568 (PMC13234497; doi:10.2196/78568)
Supplement: Checklist 2 [file jmir-v28-e78568-s003.pdf]

PRISMA-S Checklist

Section/topic	#	Checklist item	Location(s) Reported
**INFORMATION SOURCES AND METHODS**			
Database name	1	Name each individual database searched, stating the platform for each.	Methods: Search Strategy and Information Sources (PubMed (via NCBI), EMbase and Scopus (via Elsevier), Web of Science Core Collection (via Clarivate), Cochrane Central Register of Controlled Trials (CENTRAL), ProQuest Dissertations & Theses Global, and CINAHL and PsycINFO (via EBSCOhost).)
Multi-database searching	2	If databases were searched simultaneously on a single platform, state the name of the platform, listing all of the databases searched.	Methods: Search Strategy and Information Sources (No multi-database platform searching was performed; each database was searched individually.)
Study registries	3	List any study registries searched.	Methods: Search Strategy and Information Sources (ClinicalTrials.gov)
Online resources and browsing	4	Describe any online or print source purposefully searched or browsed (e.g., tables of contents, print conference proceedings, web sites), and how this was done.	Methods: Search Strategy (Section *Information Sources*) - Stated as not performed.<br>No additional online or print sources (e.g., conference proceedings, websites) were searched, as the scope of this review was limited to peer-reviewed journal articles indexed in major bibliographic databases.
Citation searching	5	Indicate whether cited references or citing references were examined, and describe any methods used for locating cited/citing references (e.g., browsing reference lists, using a citation index, setting up email alerts for references citing included studies).	Methods: Search Strategy and Information Sources (Reference lists of included studies and relevant reviews were manually screened)
Contacts	6	Indicate whether additional studies or data were sought by contacting authors, experts,	Methods: Search Strategy (Section Information Sources) - Stated as not performed.

		manufacturers, or others.	We did not contact study authors or experts for additional data, as sufficient data were available from published reports and this was not deemed necessary for the objectives of this review.
Other methods	7	Describe any additional information sources or search methods used.	Methods: Search Strategy (Section Information Sources) - Stated as not performed.<br>No additional search methods were used beyond those described, as the comprehensive database search strategy was considered sufficient to capture relevant studies.
**SEARCH STRATEGIES**			
Full search strategies	8	Include the search strategies for each database and information source, copied and pasted exactly as run.	Supplementary Table S1
Limits and restrictions	9	Specify that no limits were used, or describe any limits or restrictions applied to a search (e.g., date or time period, language, study design) and provide justification for their use.	The search was limited to English-language, peer-reviewed randomized controlled trials to ensure methodological rigor and feasibility. Language restriction was applied due to resource constraints for translation.
Search filters	10	Indicate whether published search filters were used (as originally designed or modified), and if so, cite the filter(s) used.	To ensure maximum sensitivity, no specific study design filters (e.g., RCT filters) were applied during the initial electronic database search. Instead, two reviewers independently screened all retrieved records at the title and abstract level to identify randomized controlled trials, following the Cochrane Handbook guidance.
Prior work	11	Indicate when search strategies from other literature reviews were adapted or reused for a substantive part or all of the search, citing the previous review(s).	Methods: Search Strategy (Section *Search Strategy*) - The search strategy was developed de novo and did not reuse or adapt previous reviews.
Updates	12	Report the methods used to update the search(es) (e.g., rerunning searches, email alerts).	Introduction: Rationale & Methods: Search Strategy and Information Sources (Rerunning search until March 10th, 2026). The update was

			performed by rerunning the full search strings in all identified databases to ensure the inclusion of the most recent publications.
Dates of searches	13	For each search strategy, provide the date when the last search occurred.	Methods: Search Strategy and Information Sources (March 10th, 2026)
**PEER REVIEW**			
Peer review	14	Describe any search peer review process.	The search strategy was developed and reviewed iteratively by the research team; no formal external peer review such as PRESS was conducted.
**MANAGING RECORDS**			
Total Records	15	Document the total number of records identified from each database and other information sources.	Results: Selection, inclusion, and characteristics of studies (PRISMA Flow Diagram) (Records identified from each database are reported in the flow diagram.)
Deduplication	16	Describe the processes and any software used to deduplicate records from multiple database searches and other information sources.	Records were imported into EndNote 21 for deduplication using both automated and manual processes.

PRISMA-S: An Extension to the PRISMA Statement for Reporting Literature Searches in Systematic Reviews
Rethlefsen ML, Kirtley S, Waffenschmidt S, Ayala AP, Moher D, Page MJ, Koffel JB, PRISMA-S Group.
Last updated February 27, 2020.
